# Host stimuli and operator binding sites controlling protein interactions between virulence master regulator ToxR and ToxS in *Vibrio cholerae*

**DOI:** 10.1111/mmi.14510

**Published:** 2020-04-19

**Authors:** Mareike Lembke, Thomas Höfler, Ada‐Natsuko Walter, Sarah Tutz, Vera Fengler, Stefan Schild, Joachim Reidl

**Affiliations:** ^1^ Institute of Molecular Biosciences University of Graz Graz Austria; ^2^ Division of Physiological Chemistry Medical University of Graz Graz Austria; ^3^ BioTechMed‐Graz Graz Austria; ^4^ BioHealth University of Graz Graz Austria

## Abstract

Protein–protein interactions (PPIs) are key mechanisms in the maintenance of biological regulatory networks. Herein, we characterize PPIs within ToxR and its co‐activator, ToxS, to understand the mechanisms of ToxR transcription factor activation. ToxR is a key transcription activator that is supported by ToxS for virulence gene regulation in *Vibrio cholerae*. ToxR comprises a cytoplasmic DNA‐binding domain that is linked by a transmembrane domain to a periplasmic signal receiver domain containing two cysteine residues. ToxR‐ToxR and ToxR‐ToxS PPIs were detected using an adenylate‐cyclase‐based bacterial two‐hybrid system approach in *Escherichia coli*. We found that the ToxR‐ToxR PPIs are significantly increased in response to ToxR operators, the co‐activator ToxS and bile salts. We suggest that ToxS and bile salts promote the interaction between ToxR molecules that ultimately results in dimerization. Upon binding of operators, ToxR‐ToxR PPIs are found at the highest frequency. Moreover, disulfide‐bond‐dependent interaction in the periplasm results in homodimer formation that is promoted by DNA binding. The formation of these homodimers and the associated transcriptional activity of ToxR were strongly dependent on the oxidoreductases DsbA/DsbC. These findings show that protein and non‐protein partners, that either transiently or stably interact with ToxR, fine‐tune ToxR PPIs, and its associated transcriptional activity in changing environments.

## Graphical abstract

The membrane‐bound transcription regulator ToxR controls housekeeping and virulence gene expression in *V. cholerae*. The activity and stability of ToxR strongly depends on its operon partner ToxS, the redox state of its periplasmic cysteine residues and environmental stimuli. Here, ToxR‐ToxR PPIs are the key for its activity, which is influenced by ToxS, DNA binding capabilities, bile and the disulfide bond forming enzymes DsbA/C, which induce intra‐ or intermolecular disulfide bonds.

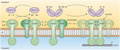

## 1 INTRODUCTION

Prokaryotes are unicellular organisms that require sensory networks for their survival in rapidly changing habitats. In the course of evolution, transmembrane signaling systems have evolved to transmit signals from the extracellular environment across the cytoplasmic membrane into the cell. One‐component signaling systems represent the oldest and simplest solution for such signal transmission, whereas two‐component systems are evolutionarily younger (Ulrich *et al.*, 2005). Although one‐component systems are widely distributed among bacteria, only 3% are directly integrated into cytoplasmic membranes (Ulrich *et al.*, 2005). A literature search revealed a non‐exhaustive list of signaling molecules that includes ToxRS, TcpPH, and TfoS in *Vibrio cholerae* and other *Vibrio* spp. (Miller *et al.*, 1987; Miller *et al.*, 1989; Hase and Mekalanos, 1998; Dalia *et al.*, 2014); CadC in *Escherichia coli* (Tetsch *et al.*, 2011); PsaE in *Yersinia tuberculosis* (Yang and Isberg, 1997); WmpR in *Pseudoalteromonas* (Stelzer *et al.*, 2006); and ArnR and ArnR1 in *Sulfolobus acidocaldarius* (Bischof *et al.*, 2019). These bitopic ToxR‐family transcription regulators consist of a single protein molecule with one input and one output domain. They share the same modular architecture—an N‐terminal winged‐helix‐turn‐helix (w‐HTH) motif located in the cytoplasm, a single inner membrane‐spanning alpha‐helical domain and a C‐terminal periplasmic signal receiver domain (Miller *et al.*, 1987; Martinez‐Hackert and Stock, 1997).

The dimerization of w‐HTH transcription factors is critical for their activation. It leads to enhanced DNA‐binding specificity and affinity, as well as increased cooperativity between the monomers (Littlefield and Nelson, 1999). The w‐HTH domain of ToxR consists of an N‐terminal ß‐sheet; three α‐helixes which include the DNA‐binding helix α3; and a C‐terminal winged helix. Interestingly, within the w‐HTH OmpR/ToxR regulator family, the ß‐sheet structure is involved in the PPIs needed for the formation of head‐to‐head or head‐to‐tail dimers (Martinez‐Hackert and Stock, 1997; Kenney, 2002; Maris *et al.*, 2005). Moreover, the wing of the w‐HTH is involved in tail‐to‐tail dimerization (Littlefield and Nelson, 1999). This was shown in HSF (heat shock transcription factor) in *Kluyveromyces lactis* using crystallography. Reports also indicate that DNA‐binding affinities are increased as a result of the activation of these one‐component transcription regulators; for example, in OmpR by N‐terminal phosphorylation. Consequently, the activated monomers bind to DNA, causing a conformational change, which, in turn, increases the affinity for a second monomer to form symmetrical or asymmetrical dimers (Rhee *et al.*, 2008).

The strongest evidence demonstrating ToxR dimerization was derived from OmpR structural studies. The dimerization may be linked to the cytoplasmic domain in which the w‐HTH motif is located. As is known in w‐HTH protein family members, dimerization via such motifs occurs due to the close localization of the monomers after their binding to the DNA operator sequences and the subsequent interaction of the N‐terminal winged helix (Littlefield and Nelson, 1999). A recent study sheds light on such mechanisms for ToxR (Morgan *et al.*, 2019). Therein, alanine‐scanning mutagenesis was performed to characterize the w‐HTH domain. Exchange mutants that lost their transcription factor activities but retained their DNA binding and possible interaction capabilities were analyzed. As a result, all characterized ToxR mutants which were identified to be transcriptionally inactive have also lost their ability to bind to DNA, including *ompU* and *toxT* operators. Although the w‐HTH region might be involved in activating transcription mechanisms, ToxR dimerization or other PPIs were not observed.

An interesting study highlighting the DNA‐dependent PPIs of CadC, a ToxR family member, in *E. coli* was recently reported by Brameyer *et al.* (2019). Such studies revealed the importance of the spatiotemporal localization and correlating transcriptional activity of CadC due to its low abundance (100 molecules per cell). They showed that activating stimuli (low pH and lysine availability) forced homodimerization and operator binding that, in turn, led to a detectable cluster formation of fluorescence labeled CadC proteins. The removal of these stimuli instantly dissolved such clusters. The authors, thereby, concluded a diffusion‐and‐capture mechanism that organizes membrane‐integrated receptors in response to DNA‐binding. Similar results have been also observed for TcpP in *V. cholerae* by single‐molecule tracking (Haas *et al.*, 2014), where both, the *toxT* promoter and ToxR, were shown to play crucial roles in TcpP motility. TcpP motility is divided into fast, slow and immobile motion behaviors. From these, it was concluded that ToxR recruits TcpP to its *toxT* promoter using a modified hand‐holding mechanism after removing nucleoid‐associated proteins (NAPs) such as H‐NS.

The dimerization of ToxR and its PPIs with other proteins, its co‐activator ToxS for instance, has long been of interest. Using the λ phage reporter system in *E. coli*, it was demonstrated that ToxR is capable of forming dimers and that ToxS seems to play a role in enhancing ToxR dimerization. In this system, the N‐terminal DNA‐binding domain of λ repressor protein C1, which lacks a C‐terminal dimerization domain, was fused to the N‐terminal cytosolic part of ToxR to assess the ability of ToxR to dimerize (Dziejman and Mekalanos, 1994). The data demonstrated that the periplasmic domain of ToxR is important for dimerization, suggesting an out‐to‐inside dimerization model facilitated by ToxS. However, the latter findings were partially rejected (Dziejman *et al.*, 1999). ToxR‐ToxS and ToxR‐ToxR PPIs were also verified using cross‐linker studies (Ottemann and Mekalanos, 1996). In these studies, ToxR homodimers were observed if ToxR was overexpressed; ToxR‐ToxS heterodimers were detected even under conditions of low expression. Moreover, in vitro analysis using purified periplasmic domains of ToxS and ToxR led to the identification of ToxR‐ToxS PPIs by utilizing NMR and reciprocal pull‐down assays (Midgett *et al.*, 2017).

In *V. cholerae*, ToxRS has emerged as a key regulatory complex involved in virulence gene regulation. The transmembrane spanning domains of ToxRS offer unique possibilities for perceiving and transducing signals into transcriptional regulation programes. Some activating conditions and substances were identified as bile salts, alkaline pH, and nutrient availability (Matson *et al.*, 2007; Childers and Klose, 2007; Peterson and Gellings, 2018). Despite its important role for virulence and environmental adaption, the exact mechanism of ToxR signal transduction and transcription factor activation remains to be characterized. Many studies, summarized above and recently published by Morgan *et al.* (2019), showed evidence for ToxR dimerization. However, no detailed information about the interaction interface and orientation is available. Insights about the complexity of the ToxR family protein activation have been derived from an analysis of cysteine‐based intra‐ and intermolecular disulfide bond formations in the periplasm. Some examples include bile salt (taurocholate)‐induced intermolecular disulfide bond formation and activation in TcpP (Yang *et al.*, 2013) or cysteine‐dependent, intermolecular heterodimeric interactions of TcpP and ToxR under anaerobic conditions and subsequent virulence gene activation via *toxT* transcription (Fan *et al.*, 2014). Finally, the cysteine residues in ToxR are associated with its transcriptional activity through intramolecular disulfide bond formation. Moreover, they also provide a signal for proteolysis once they appear in their reduced form (Ottemann and Mekalanos, 1996; Fengler *et al.*, 2012; Lembke *et al.*, 2018).

In summary, information on the interplay between ToxRS molecules remains fragmented and incomplete. In this study, we focus on ToxR PPIs and its known interaction factors. We found that ToxR‐ToxR PPIs were enhanced in the presence of ToxR operator binding sites, ToxS and bile. Additionally, ToxR‐ToxS PPIs were detected using an adenylate‐cyclase‐based bacterial two‐hybrid system in *E. coli*. Finally, we extend our previous model by showing that the intermolecular disulfide bond formation of ToxR periplasmic domains is DsbA/DsbC‐dependent in *V. cholerae*, and that formation of this homodimer is associated with enhanced transcriptional activity.

## 2 RESULTS

### 2.1 ToxS, DNA operator binding sites, and bile enhance ToxR PPIs

**FIGURE 1:**
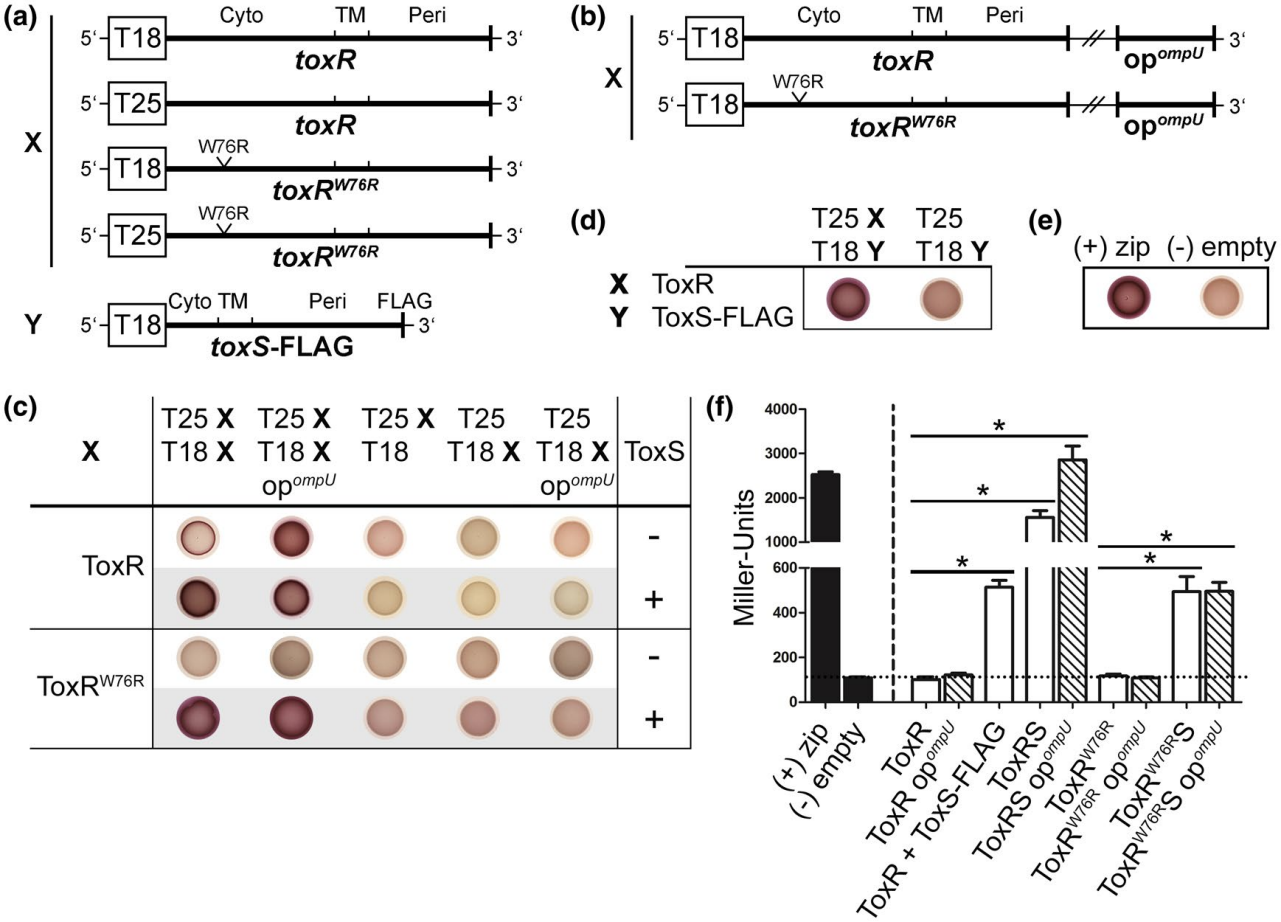
ToxS and *ompU* operator binding sites are key players in ToxR PPIs in *E. coli*. PPIs of the indicated ToxR and ToxS‐FLAG variants were tested in *E. coli* W3110 ∆*cyaA* using a bacterial cAMP‐based two‐hybrid system (BACTH), which is based on the functional complementation of the adenylate cyclase CyaA (Karimova *et al.*, 1998). Strains are N‐terminal ToxR, ToxR^W76R^ and ToxS‐FLAG translational fusions linked to the C‐termini of the *B. pertussis* CyaA T18 or T25 domains with or without *V. cholerae ompU* operator fragments (op*^ompU^*), and co‐expressed ToxS (a, b). The leucine zipper of the yeast GCN4 protein (zip) was used as a positive complementation control (+), while the empty plasmids pKT25 and pUT18C served as negative controls (‐) (e, f). For the drop test (c, d, e), strains were grown in LB overnight and subsequently transferred to a single MacConkey maltose indicator plate to reveal the CyaA^+^ phenotype (red colonies indicate the utilization of maltose as a C‐source). PPIs are shown between the indicated ToxR or ToxR^W76R^ translational fusions (designated as X) in the presence or absence of co‐expressed ToxS and *ompU* operator binding sites (op^ompU^) (c). Panel (d) displays PPIs between ToxR and ToxS‐FLAG (designated as Y). Panel (f) shows quantifications of functional complementation between the indicated ToxR, ToxR^W76R^ and ToxS‐FLAG hybrid proteins (white bars) in dependence of *ompU* operator binding sites (op^ompU^, lined white bars) by measuring β‐galactosidase activities. The cells were grown in LB supplemented with 0.05 mM IPTG to the stationary phase. Strains in which ToxR and ToxR^W76R^ PPIs were measured in the presence of *c*o‐expressed ToxS were labeled with ToxRS or ToxR^W76R^S. Strains in which interactions between ToxR and ToxS‐FLAG were analyzed were labeled with ToxR + ToxS‐FLAG. The positive and negative controls are represented by black bars. The values are means of three biological replicates, each with technical triplicates with error bars, which represent the standard deviation. Interactions are reported as Miller Units. The asterisks indicate significantly different means with *p* < .05 for the respective columns, each tested against *E. coli* W3110 ∆*cyaA* pKT25‐ToxR pUT18C‐ToxR or pKT25‐ToxR^W76R^ put18C‐ToxR^W76R^ using one‐way ANOVA test with Bonferroni post hoc analysis [Colour figure can be viewed at wileyonlinelibrary.com]

Transcription regulators containing w‐HTH domains rarely act by themselves but form dimers to induce specific cellular responses (Littlefield and Nelson, 1999). More than 30 years ago, it was postulated that ToxR either acts as a homodimer (Miller *et al.*, 1987; Dziejman and Mekalanos, 1994; Ottemann and Mekalanos, 1996) or in cooperation with other proteins (DiRita and Mekalanos, 1991; Krukonis *et al.*, 2000). However, the molecular mechanism behind ToxR PPIs and its activity is still poorly understood. To dissect the roles of ToxS, DNA operator binding sites, and environmental stimuli such as bile, in ToxR PPIs, a bacterial cAMP‐based two‐hybrid system (BACTH) (Karimova *et al.*, 1998) was used in *E. coli* W3110 ∆*cyaA*. The BACTH system is accessible to membrane proteins and is based on the reconstitution of the T25 and T18 domains of the adenylate cyclase CyaA from *Bordetella pertussis*, resulting in cAMP synthesis. In our experiments, the N‐termini of potentially interacting proteins were fused to the C‐termini of the two CyaA fragments because of their predetermined orientation in the inner membrane (Figure 1a,b). The respective fusion proteins were tested separately for the expression in *E. coli* XL1‐Blue, DH5α λpir and BL21 (DE3) (Figure S1). For the investigation of putative ToxR‐ToxR and ToxR‐ToxS PPIs, *E. coli* strain W3110 ∆*cyaA* was co‐transformed with the combinations of pUT18C and pKT25 derivatives carrying translational fusions of T18‐ToxR/T25‐ToxR; T18‐ToxR^W76R^/T25‐ToxR^W76R^, co‐expressed with or without ToxS, respectively, and T25‐ToxR/T18‐ToxS‐FLAG.

Next, protein interactions were tested by spotting the resulting *E. coli* strains on MacConkey maltose agar plates (Figure 1c,d,e) and by measuring β‐galactosidase activities (Figure 1f). Positive interactions that generated an elevated adenylate cyclase activity were detected as red colonies on MacConkey maltose agar plates or through the increased expression of the *lacZ* reporter. We chose a cut‐off value of 100 Miller Units (Figure 1f), predetermined by the negative control, as indicative of a false positive interaction between the fusion proteins. This approach demonstrated that the co‐expression of ToxS with ToxR or the DNA‐binding‐deficient mutant ToxR^W76R^, resulted in a red colony phenotype and significantly increased *lacZ* expression levels compared to strains without ToxS (Figure 1c,f). Strains expressing ToxR or ToxR^W76R^ alone displayed a white colony phenotype and Miller Units below or equal to the cut‐off level. Thus, we found that ToxR‐ToxR PPIs were enhanced in the presence of its operon partner ToxS.

Since ToxS was able to mediate ToxR‐ToxR PPIs, we were also interested in the interaction of ToxR with ToxS. Here, we were able to confirm ToxR‐ToxS‐FLAG PPIs using BACTH (Figure 1d,f), which, in turn, emphasizes the results of earlier studies by Midgett *et al.* (2017).

ToxR is a transcriptional regulator located in the inner membrane and binds to its operator binding sites after activation. Therefore, we also addressed the question of whether the *ompU* operator binding sites, also termed ToxR boxes, capture ToxR molecules to result in ToxR‐ToxR PPIs. Based on the direct repeat nature of ToxR‐binding sites in the *ompU* promoter region (5′‐TNAAA‐N5‐TNAAT‐3ʹ), located from −51 to −37 relative to the transcription start site (Goss *et al.*, 2013), we suggest a cooperative binding of two ToxR molecules. To test our hypothesis, the *V. cholerae ompU* operator fragment (op*^ompU^*) (Morgan *et al.*, 2011) was cloned into pUT18C to provide ToxR with its natural DNA‐binding‐sites in *E. coli* (Figure 1b). Interestingly, the red colony phenotype (Figure 1c) indicated that the presence of the *ompU* operator binding sites triggered ToxR‐ToxR PPIs independently of ToxS. However, this could not be confirmed by the β‐galactosidase assay (Figure 1f). This implied that the MacConkey maltose agar plates exhibit a higher sensitivity for the evaluation of PPIs, which remains to be elucidated. Nevertheless, the ToxR‐ToxR PPIs were significantly increased in the strains that co‐expressed ToxS and provided *ompU* operators compared to those strains without *ompU* operators. In contrast, the *ompU* operators showed no effect on the ToxR^W76R^ DNA‐binding‐deficient mutant with or without ToxS (Figure 1c,f). Thereby, we emphasize our above findings that ToxR‐boxes play a major role in ToxR‐ToxR PPIs, especially in the presence of ToxS.

**FIGURE 2:**
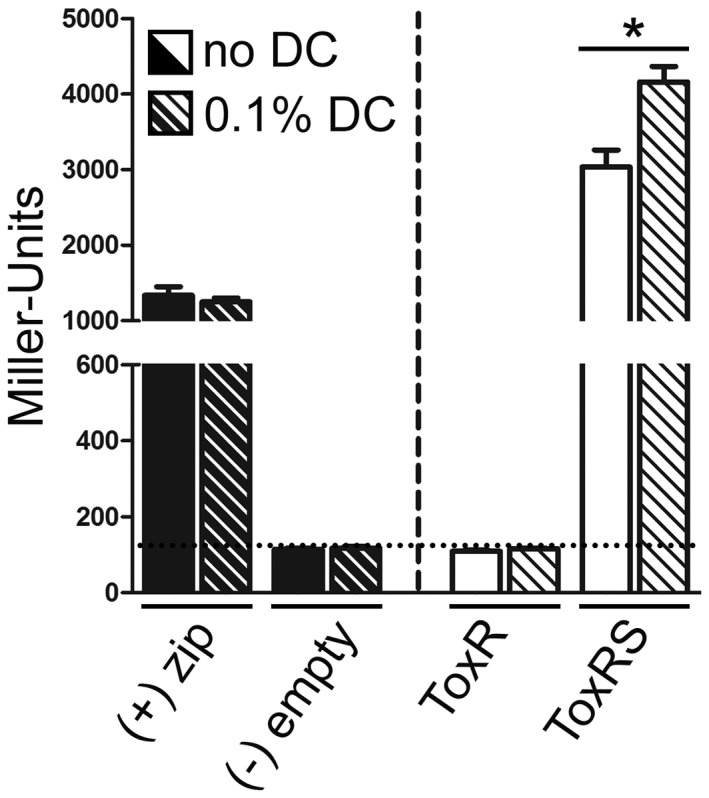
Bile salts (DC) stimulate ToxR PPIs in a ToxS dependent manner. PPIs of ToxR with or without co‐expressed ToxS were tested in dependence of bile salts in *E. coli* W3110 ∆*cya*A using BACTH (Karimova *et al.*, 1998). See experimental procedures for details. The leucine zipper of the yeast GCN4 protein (zip) was used as a positive complementation control (+), while the empty plasmids pKT25 and pUT18C served as negative controls (‐). Cells were grown in LB supplemented with 0.05 mM IPTG to the stationary phase in the absence or presence of 0.1% DC (sodium deoxycholate). The functional complementation of the controls (black bars) and ToxR with or without ToxS (white bars) was quantified by measuring β‐galactosidase activities. DC treated samples are indicated by lined bars. The values are means of three biological replicates, each with technical triplicates with error bars, which represent the standard deviation. The controls contain five values each. Interactions are reported as Miller Units. The asterisks indicate significantly different means with *p* < .05 using Student's *t*‐test

During infection, bacterial pathogens of the small intestine are surrounded by adverse conditions, including bile salts that circulate between the intestine and the liver of vertebrates (Hofmann *et al.*, 2010). Therefore, we tested whether incubation with the bile salt sodium deoxycholate (DC) has an impact on ToxR‐ToxR PPIs (Figure 2). Our results demonstrated that if *toxS* was co‐expressed, the addition of 0.1% DC increased PPIs between ToxR molecules. This indicates that DC represents a trigger factor that facilitates ToxR PPIs in dependence of ToxS. In contrast, the leucine zipper positive and negative controls did not respond to bile.

Taken together, we show that the membrane‐bound transcription regulator ToxR exhibited dynamic interaction states. In *E. coli*, the ToxR‐ToxR PPIs of the cytoplasmic domains were mediated by ToxS. These ToxR interactions were further enhanced by *ompU* operators provided on a plasmid or bile added into the growth media. Our results may indicate a hierarchical order in the generation of a functional ToxRS complex. The first step involves contact with ToxS, leading to significantly increased ToxR‐ToxR PPIs. Next, we observed that ToxR‐ToxR PPIs can further be stimulated in the presence of bile, but only if ToxS was present. Finally, ToxR operators capture preliminary formed ToxRS complexes leading to the highest ToxR‐ToxR PPI values measured.

### 2.2 ToxR transcription factor activity correlates with the formation of homodimers

**FIGURE 3:**
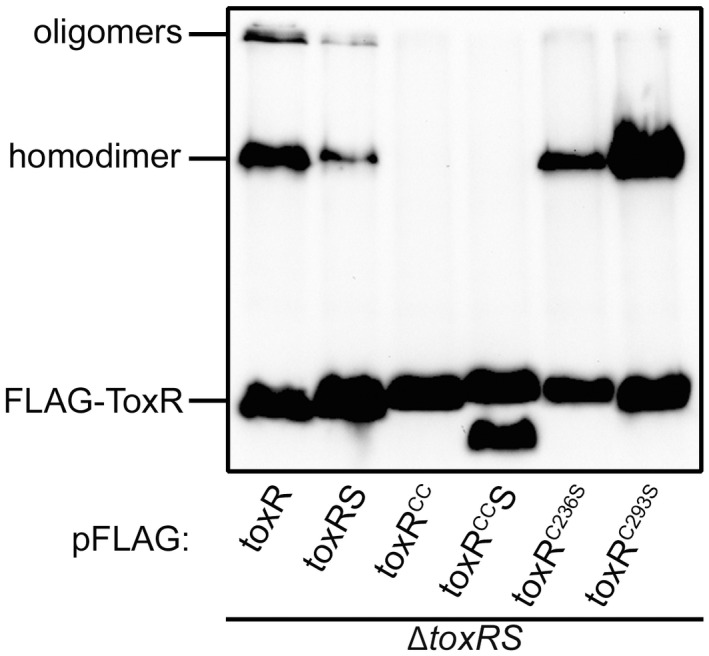
Cysteine dependent homodimer formation in ToxR. *V. cholerae* Δ*toxRS* strains carrying *toxR* derivatives with or without its operon partner *toxS* on pFLAG‐MAC^TM^ were grown in LB. Samples were taken after 2 hr induction with 0.05 mM IPTG in the mid‐log phase and analyzed by immunoblotting using anti‐FLAG antibodies. Immunoblots were carried out under standard non‐reducing Laemmli buffer conditions

Based on the abovementioned observations, we focused on PPIs taking place in the periplasmic domain of ToxR, namely by characterising disulfide bond formations and their influence on dimerization and activity. We recently demonstrated that the two cysteine residues in the periplasmic domain are responsible for the maintenance of ToxR stability and activity (Lembke *et al.*, 2018). To find a connection between ToxR activity and homodimer formation, we focused on ToxR cysteine residues. To this end, native *toxR* or cysteine mutants (C236S, C293S or CC, the latter is an exchange of both cysteine residues with serine) were cloned into pFLAG‐MAC^TM^ under *tac* promoter expression control. As previously mentioned, we also cloned the operon partner gene *toxS* into the plasmids. When introduced into *V. cholerae* Δ*toxRS*, the monomeric, dimeric, and oligomeric forms of FLAG‐tagged ToxR derivatives were analyzed by SDS‐PAGE and immunoblotting under non‐reducing conditions (Figure 3, see respective loading control in Figure S2). There, the disulfide bond‐dependent homodimerization and oligomerization of FLAG‐ToxR was decreased in the presence of ToxS, suggesting that ToxS competes for interactions with ToxR molecules for disulfide bond formation in the periplasm. The FLAG‐ToxR^CC^ mutants lacking both cysteines showed a complete loss of the ability to form homodimers, which was independent of ToxS. Therefore, intermolecular disulfide bonds were responsible for the observed PPIs. To note, a proteolytic FLAG‐ToxR^CC^ degradation fragment was observed when ToxS was co‐expressed. However, the ability of ToxR to form homodimers was restored in the FLAG‐ToxR^C236S^ and FLAG‐ToxR^C293S^ single cysteine mutants. Strikingly, high levels of homodimers were observed for the FLAG‐ToxR^C293S^ mutant, indicating that the altered thiol redox state of Cys293 favored such dimer formations (Figure 3). These data also demonstrated that the periplasmic cysteine residues were close enough to form intermolecular disulfide bonds to yield homodimers.

To determine the correlation between homodimer formation and ToxR activity, we monitored the PhoA activities and OmpU/T protein levels in parallel using strains with chromosomal *ompU*::*phoA* and *ompT*::*phoA* fusions. The *ompU* and *ompT* expression levels provide an excellent readout for ToxR activity, as they are inversely regulated by ToxR (Crawford *et al.*, 1998; Li *et al.*, 2000). We previously reported that the ToxR^CC^ cysteine mutant is a target for regulated intramembrane proteolysis (RIP) (Lembke *et al.*, 2018). We now show that RIP not only affected the ToxR^CC^ mutant but also the single cysteine mutants when grown in M9 maltose minimal medium (Figure S3). Therefore, this experiment was carried out in a Δ*degP* background in the mid‐log phase to ensure similar ToxR protein levels for ToxR^WT^ and the cysteine mutants to allow a comparison of PhoA activities between different proteolysis prone *toxR* mutants (Figure S4a,b).

**FIGURE 4:**
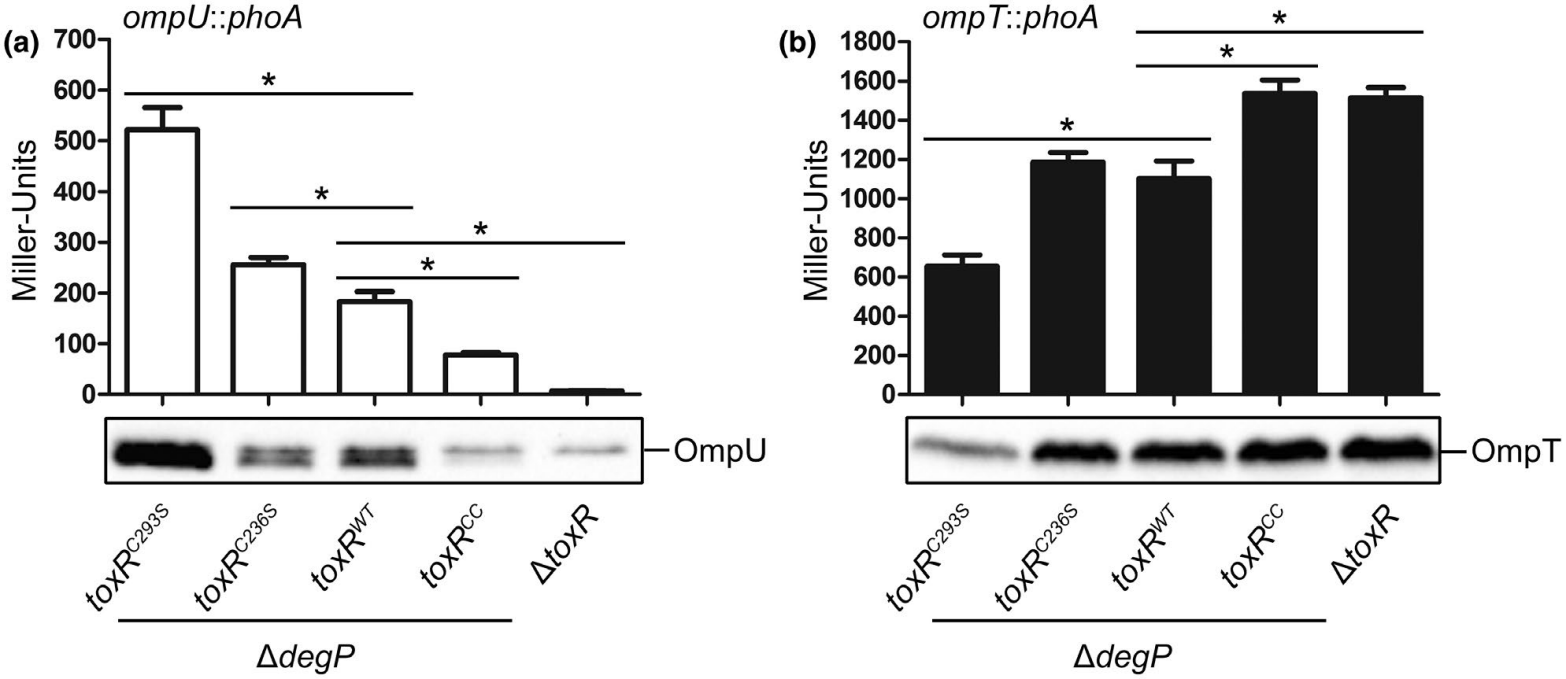
Transcription factor activity of *toxR* cysteine mutants. Shown are reporter gene activities of alkaline phosphatase PhoA (Miller Units) linked as operon fusions to either (a) *ompU* (white bars) or (b) *ompT* (black bars) in *V. cholerae* Δ*degP* strains harboring various chromosomal cysteine mutations in *toxR*. Simultaneously, immunoblot analysis was performed under standard reducing Laemmli buffer conditions to detect OmpU or OmpT, respectively. Cells were grown in M9 maltose minimal medium and samples were taken in the mid‐log phase. *V. cholerae* Δ*toxR* served as a negative control. The mean values with standard deviation are shown (*n* = 6). The asterisks indicate significantly different means with *p* < .05 for the respective columns each tested against Δ*degP toxR^WT^* using one‐way ANOVA test, followed by Dunnett's post hoc test for multiple comparisons. To note, all the characterized strains featured a chromosomally *toxS^+^* background

When grown in M9 maltose minimal medium, the strain expressing ToxR^WT^ exhibited a more pronounced *ompT* expression compared to *ompU* (Figure 4a,b). As expected, the Δ*toxR* control showed neither activated *ompU* transcription nor *ompT* repression. For comparison, chromosomal *toxR* cysteine mutants, constructed by exchanging one or both cysteines to serines (C236S, C293S or CC), were also analyzed. There, as expected, the *toxR^CC^* strain displayed significant regulatory deficiencies for *ompU* and *ompT* expression when compared to *toxR^WT^*, as we have previously shown (Lembke *et al.*, 2018). Thus, this indicates a possible link between disulfide bond formation and ToxR activity. In contrast, the ToxR^C236S^ mutant was able to activate *ompU* beyond the strain expressing ToxR^WT^, although simultaneous *ompT* repression seemed to be less evident. In particular, the ToxR^C293S^ replacement mutant strongly activated *ompU* and repressed *ompT* significantly beyond the strain expressing ToxR^WT^. To be mentioned, this happened despite growing the strains under nutrient‐limiting conditions that do not favor ToxR activation. In addition, OmpU and OmpT protein expression patterns, which were detected by immunoblot analysis from the same cultures (Figure 4a,b), showed similar results to the PhoA activity measurements. To note, all the characterized strains featured a chromosomally *toxS^+^* background.

Taken together, these results indicate that ToxR cysteine residues contribute to the transcriptional activity of ToxR, presumably because they are required for intra‐ and intermolecular disulfide bond formation. Moreover, we conclude that cysteine‐dependent transcription factor activity correlates with the formation of homodimers, supporting the early view by Miller *et al.* (1987).

### 2.3 DNA‐binding triggers ToxR homodimer formation

Our results thus far suggest that ToxR homodimerization strongly correlates with its activation, ultimately resulting in the transcriptional regulation of genes such as *ompU* and *ompT*. These findings raised the question of which factors or conditions influence the ToxR‐ToxR PPIs. The DNA‐binding domains of the OmpR family proteins generally facilitate dimer formation once they are in contact with direct repeat DNA sequences (Yoshida *et al.*, 2006). We, thus, investigated the effect of operator binding on ToxR dimerization in more detail in *V. cholerae* to expand the data derived from our BACTH analysis in *E. coli*.

**FIGURE 5:**
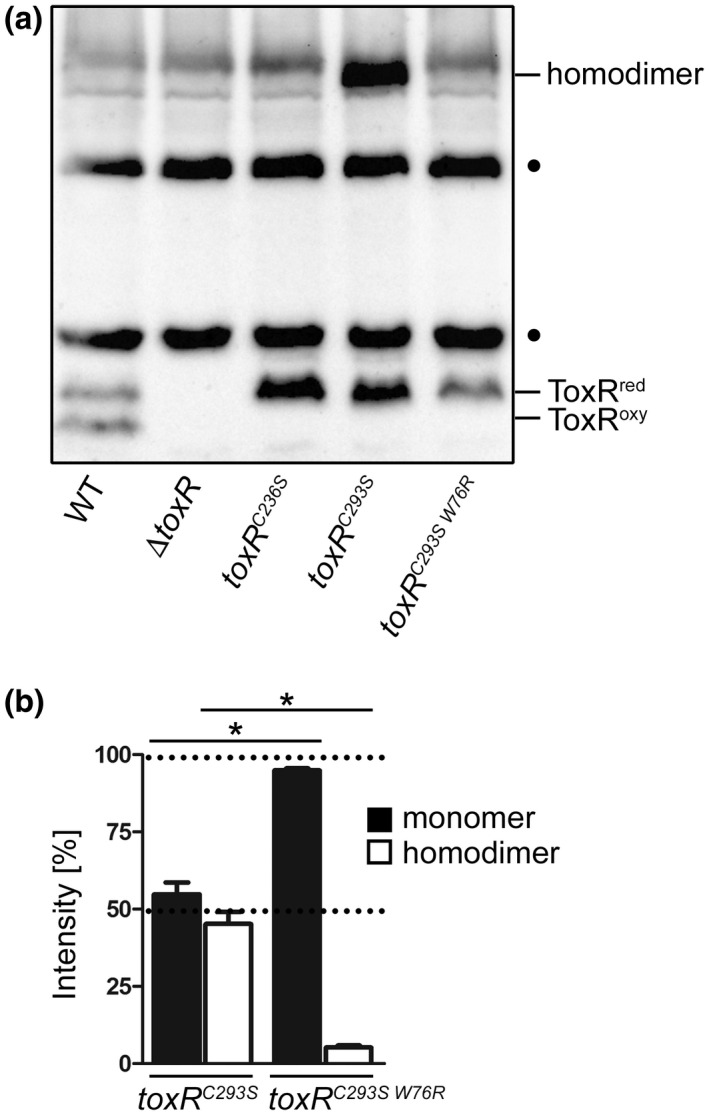
DNA binding triggers ToxR homodimer formation. (a) Shown is ToxR immunoblot analysis of *V. cholerae* WT, Δ*toxR*, *toxR^C236S^*, *toxR^C293S^*, and *toxR^C293SW76R^* grown in M9 maltose minimal medium until the mid‐log phase was reached. Immunoblotting was performed under standard non‐reducing Laemmli buffer conditions using anti‐ToxR antibodies. (•) Represents nonspecific cross‐reacting background bands. (b) The column bar graph displays the protein band intensities of ToxR monomers (black bars) and homodimers (white bars) in *V. cholerae toxR^C293S^* compared to *toxR^C293SW76R^* as a result of densitometric analysis carried out under non‐reducing Laemmli buffer conditions (see representative immunoblot Figure S5a). Here, ToxR protein band intensities were measured per strain (both intensities add up to 100%) using Image Lab Software (BIO‐RAD). The mean values with standard deviation are shown (*n* = 6). The asterisks indicate significantly different means between *toxR^C293S^* and *toxR^C293SW76R^* monomers and homodimers with *p* < .05, respectively, using Student's *t*‐test. To note, all the characterized strains featured a chromosomally *toxS^+^* background

The WT or single cysteine replacement mutants were grown in M9 maltose minimal medium to express the reduced (ToxR^red^) and oxidized (ToxR^oxy^) monomeric and dimeric forms of chromosomally encoded *toxR*. These were detected by SDS‐PAGE and immunoblotting under non‐reducing conditions (Figure 5a). To note, the following experiments have been carried out in a *degP^+^* background to avoid distortions leading to artificial *toxR* expression patterns under non‐reducing conditions. Compared to the WT control, only the reduced monomeric form of ToxR was detected in the *toxR* single cysteine mutants, as they were unable to form intramolecular disulfide bonds (Ottemann and Mekalanos, 1996; Lembke *et al.*, 2018). Notably, ToxR homodimers could only be detected in the *toxR^C293S^* mutant but not in the WT or the *toxR^C236S^* mutant in the various growth conditions tested (LB, M9 glucose or maltose minimal medium with and without NRES, AKI or media with bile salt supplementation) (data not shown). Since the addition of reducing agents in Laemmli buffer (β‐mercaptoethanol) (Laemmli, 1970) dissolved ToxR^C293S^ homodimers, we deduced that these homodimers were formed by intermolecular disulfide bonds (compare Figure 5a with Figure S3). To determine the impact of DNA binding on ToxR‐ToxR PPIs, a W76R point mutation (according to the amino acid position as annotated by Heidelberg *et al.* (2000)) in the w‐HTH domain of ToxR^C293S^ was introduced (resulting in ToxR^C293SW76R^). This amino acid substitution was first described by Morgan *et al.* (2011) as a mutation that is detrimental for DNA binding and activation of the *ompU* and *toxT* promoters. As presented in Figure 5a, the removal of operator binding abilities in ToxR^C293SW76R^ consequently resulted in undetectable homodimer formation. This indicates that ToxR‐boxes may serve as an anchor point for PPIs between ToxR molecules, for example, homodimers. Since the *toxR* expression levels varied between *toxR^C293S^* and *toxR^C293SW76R^* strains, a more precise quantification analysis was performed to verify the impact of ToxR‐boxes on ToxR homodimerization. Shown in Figure 5b are the results of densitometric analysis (Figure S5). Data were calculated as absolute values of intensity per lane and sample and expressed as a percentage of the sum of both (homodimer and monomer) intensities. As a result, we saw a higher proportion of monomer relative to dimer formation for ToxR^C293SW76R^ compared to ToxR^C293S^. These results indicate that loss of dimer formation correlates with the inability to bind to ToxR‐boxes. In summary, these results reveal a capture mechanism that organizes ToxR in the presence of operator sites to form cysteine‐dependent homodimers.

### 2.4 DsbA and DsbC coordinate intra‐ and intermolecular disulfide bond formation in ToxR

Disulfide bonds are formed by the oxidation of two cysteine residues in close proximity, for example, 2.5 Å (Overington *et al.*, 1992). This reaction can proceed spontaneously or with the help of enzymatic catalysts. Many secretory proteins, such as cholera toxin (Tomasi *et al.*, 1979), undergo oxidative folding, in which they acquire intra‐ or intermolecular disulfide bonds to form higher‐order quaternary structures. The periplasmic space of Gram‐negative bacteria contains multiple disulfide bond‐forming enzymes, for example, DsbABCD, which catalyze the formation and isomerization of disulfide bonds. Since disulfide bond formation plays an essential role in ToxR activity and its homodimerization, Dsb proteins were studied in greater detail.

**FIGURE 6:**
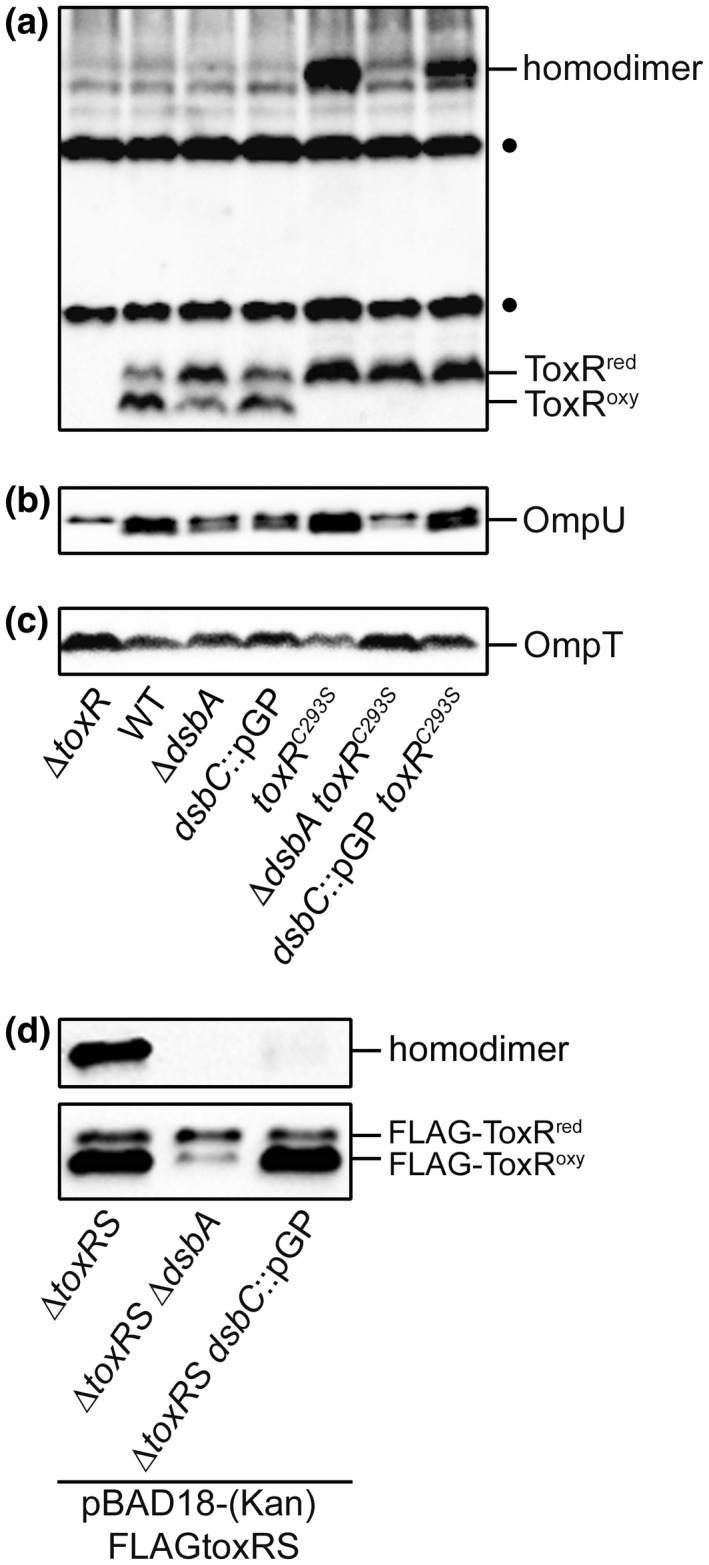
Dsb proteins influence ToxR inter‐ and intramolecular disulfide bond formation and its activity. *V. cholerae* strains harboring *dsbA*, *dsbC* and/or *toxR* mutations were grown in M9 maltose minimal medium until the mid‐log phase was reached. For Δ*toxRS* strains carrying FLAG*toxRS* on pBAD18‐Kan or pBAD18, samples were taken after 2 hr of induction with 0.1% arabinose when the cells reached the mid‐log phase. Immunoblotting was performed under standard non‐reducing Laemmli buffer conditions using anti‐ToxR (a) or anti‐FLAG antibodies (d); or reducing conditions using anti‐OmpU (b) or anti‐OmpT antibodies (c). (•) Represents nonspecific cross‐reacting background bands. It is to note, that Kang‐gel staining was performed to provide similar protein levels of all samples shown (Figure S6a,b)*.* To note, all the characterized strains featured a chromosomally *toxS^+^* background, except for the Δ*toxRS* strain used

Cells were grown in M9 maltose minimal medium harboring mutations in the thiol‐disulfide oxidoreductase *dsbA* or the disulfide bond isomerase *dsbC* (Missiakas *et al.*, 1995; Kadokura *et al.*, 2003). SDS‐PAGE and immunoblotting were performed under non‐reducing conditions to expose the redox state of ToxR (Figure 6a,d), as well as its activation state, by monitoring OmpU (Figure 6b) and OmpT (Figure 6c) protein levels. For loading controls, see supplemental data (Figure S6). Furthermore, using densitometric analyses (Figure S7), we quantified the synthesis of OmpU/T. To note is that the ratio observed for ToxR^red/oxy^ (see Figures 5 and 6) can be variable, depending on culture conditions and sample handling. Therefore, comparisons between different mutants always require the usage of the same culture media and growth conditions, best applied along with the same series of the experiment.

As was observed for chromosomal ToxR^WT^ (Figure 6a–c), mutations in *dsbA* or *dsbC* significantly decreased OmpU but had no effect on OmpT protein levels (Figure S7) compared to the WT. This demonstrates a loss of ToxR activity due to the absence of Dsb proteins, especially for the *ompU* transcription activation. The overexpression of FLAG‐ToxRS revealed that the decreased activity of chromosomal ToxR^WT^ in *dsbA* and *dsbC* mutants (Figure 6b,c) correlates with decreased homodimer formation (Figure 6d). It is to note that homodimer formation was not observed if ToxRS was expressed from chromosomally encoded loci but was readily detected if *toxRS* were overexpressed by the pBAD expression system (compare Figure 6a,d). Interestingly, there were no observable changes in the redox state of monomeric ToxR^WT^ in the *dsbC* mutant compared to the WT (Figure 6a), indicating no interference in the redox equilibrium of the monomeric form. However, intramolecular disulfide bond formation in ToxR^WT^ was disturbed in a *dsbA* mutant strain, as was shown previously (Lembke *et al.*, 2018). In the study, the amount of monomeric ToxR^red^ was higher than that of ToxR^oxy^. As an extension of our previous model, we suggest that DsbA introduces intramolecular disulfide bonds into newly translated ToxR polypeptides (Lembke *et al.*, 2018). Only the monomeric, oxidized ToxR molecule (ToxR^oxy^) represents a substrate for the isomerase DsbC, which achieves the native disulfide proteome of the cell (Missiakas *et al.*, 1995; Kadokura *et al.*, 2003).

To decipher disulfide bond formations in *toxR^C293S^*, mutations in *dsbA* and *dsbC* were also introduced here. In particular, the *toxR^C293S^* mutant was able to activate *ompU* and repress *ompT* transcription beyond ToxR^WT^ levels (Figure 4a,b) when grown in the M9 maltose minimal medium. In contrast to ToxR^WT^, ToxR^C293S^ only possessed the option to form intermolecular disulfide bonds. The serine substitution of one of the two cysteine residues did not enable intramolecular disulfide bond formation but instead resulted in the monomeric ToxR^red^ and the homodimeric form (Figure 6a). Changes in the redox status of ToxR^C293S^ in the *dsb* mutants were, therefore, only detectable in the homodimers. As shown in the *dsbA* mutant, homodimer formation was abolished (Figure 6a) and the decreased activity of ToxR^C293S^ became apparent for OmpU and OmpT expression (Figures 6b,c, S7). The insertion in *dsbC* had less impact on both porin expression levels, presumably because intramolecular disulfide bonds, which serve as DsbC substrates, cannot be formed in ToxR^C293S^.

Taken together, these results allowed us to confirm that the ToxR cysteine residues are critical for its activation state. Furthermore, we postulate that the transcriptional activity of ToxR correlates with the formation or interplay of cysteine‐dependent homodimers and that DsbA and DsbC contribute to this specific ToxR folding.

## 3 DISCUSSION

Only a minority of the one‐component systems are directly integrated into cytoplasmic membranes. Because more than 80% of the signal transduction pathways involve the binding of DNA, an arrangement of membrane‐bound signal transducers may place major constraints on their ability to interact with DNA (Ulrich *et al.*, 2005; Jung *et al.*, 2018). ToxR is one such membrane‐bound one‐component signal transducer that is required for *V. cholerae’s* lifestyle switch between the host and the environment. The dimerization of transcription factors often leads to enhanced DNA‐binding specificity and affinity—characteristics that mitigate the constraints on ToxR–DNA interactions (Littlefield and Nelson, 1999). In this study, we addressed how ToxR may overcome the difficulties that it experiences as a membrane‐bound transcriptional regulator by forming dynamic PPIs that depend on DNA operators, co‐activator ToxS, ToxR‐cysteine residues, Dsb mediated activities and ToxR activating stimuli (e.g., bile, DC). Therefore, we particularly focused on housekeeping genes (OmpU/T), since the cysteine residues seem to play an important role in their regulation (Fengler *et al.*, 2012; Lembke *et al.*, 2018).

**FIGURE 7:**
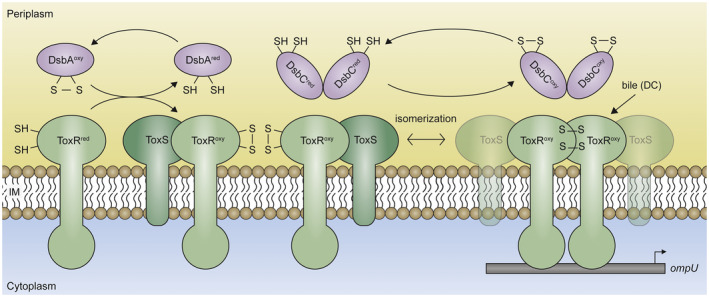
Activation mode and interaction patterns of ToxRS. Integral membrane proteins such as ToxRS are inserted into the cytoplasmic membrane co‐translationally, whereby ToxR exposes its reduced cysteine residues to the periplasm. These cysteines are then oxidized by DsbA to form intramolecular disulfide bonds, which represent the proteolytically stable form of ToxR. Increased ToxR‐ToxR PPIs can be detected if ToxS and ToxR‐boxes (e.g., *ompU* promoter) are available. Such an interaction is further strengthened in the presence of bile (sodium deoxycholate). As a model, we suggest that while ToxR molecules are bound to their operator sequences (ToxR‐boxes), temporarily formed ToxR dimers may exist due to intermolecular disulfide bond formation depending on DsbC or other mechanisms [Colour figure can be viewed at wileyonlinelibrary.com]

The presence of direct repeat DNA sequences in operators, similar to OmpR operators in *E. coli* (Yoshida *et al.*, 2006), recognized by ToxR (ToxR‐boxes) argues for the binding of ToxR dimers (Goss *et al.*, 2013). Here, we demonstrate that ToxR DNA binding enhances ToxR‐ToxR PPIs and dimer formation. For example, we show in *V. cholerae* that the number of disulfide‐linked homodimers of a chromosomal ToxR^C293S^ variant was significantly decreased once the protein was unable to bind its operators after the introduction of a W76R mutation in its w‐HTH domain (Morgan *et al.*, 2011). These results were further supported by a bacterial cAMP‐based two‐hybrid system (BACTH) in *E. coli*. There, the presence of plasmid‐encoded *V. cholerae ompU* operator binding sites enhanced ToxR‐ToxR PPIs when ToxS was co‐expressed. To note, in the absence of co‐activator ToxS, the efficiency of interactions between these ToxR fusion proteins was not particularly strong. In comparison, the ToxR^W76R^ operator‐binding‐deficient mutant displayed no enhancement of PPIs in the presence of the *ompU* operator‐binding sites. At this point, we propose that ToxR DNA operators may serve as an anchor point for the subsequent formation of ToxR‐ToxR PPIs and these interactions are further enhanced in the presence of ToxS (Figure 7). Brameyer *et al.* recently described a similar mechanism in *E. coli* where the ToxR‐like membrane‐bound transcriptional regulator CadC formed PPIs when external stresses activated the receptor which ultimately resulted in DNA binding (Brameyer *et al.*, 2019). Owing to their membrane‐anchoring, ToxR‐like transcription regulators are limited in their spatial dynamics. However, the formation of homodimers may support these regulators to tether DNA close to the cytoplasmic membrane.

The model of a dynamic ToxR monomer and dimer formation derived from earlier studies suggests that the inactive form of ToxR is a monomer and the active one is a dimer. This was shown using ToxR‐PhoA fusions proteins or cross‐linking techniques, respectively (Miller *et al.*, 1987; Ottemann and Mekalanos, 1996). However, an inconclusive picture regarding the ToxR localization and dimerization status still exists. The exact conditions necessary for the regulation of *ompU/T* are not known—both, cytosolic and soluble or membrane‐bound forms of ToxR were found to be sufficient for *ompU/T* regulation and TcpP controlled *toxT* regulation, independently of its periplasmic domain (Crawford *et al*., 2007). Moreover, other studies indicate that a cytosolic, soluble ToxR or ToxR periplasmic truncations only promote *ctxAB* gene expression in *E. coli* when fused to dimerization domains (e.g., Leu‐zipper). However, such constructs failed to show activity in *V. cholerae* (Dziejman *et al.*, 1999). Furthermore, it was shown that PPIs also take place between TcpP and ToxR and that such interactions involve cysteine‐dependent disulfide bond formation in their periplasmic domains and anoxic growth conditions (Fan *et al.*, 2014). Our data add to the current knowledge by showing that periplasmic cysteine residues interconnect ToxR molecules by disulfide bond formations via Dsb‐enzymes which plays a major role in ToxR transcriptional activity. The replacement of both cysteine residues with serine decreases ToxR activity. This was best demonstrated by monitoring *ompU* and *ompT* transcription in various cysteine mutants. Although the ToxR single cysteine mutants did not have the potential to form intramolecular disulfide bonds, these mutants were found to form homodimers. Interestingly, data obtained using Cys293 or Cys236 mutants demonstrated that periplasmic ToxR domains must come into immediate contact with each other in order to form intermolecular disulfide bonds. In addition, such close contact of the periplasm localized cysteine residues was highly dependent on the ability of ToxR to bind its operators, for example, *ompU* operators, as if there was signaling from the inside to the outside. Furthermore, both single cysteine ToxR mutants were able to activate *ompU* transcription beyond native ToxR levels in minimal medium. It should be emphasized, however, that the sole ability of ToxR homodimer formation does not automatically correlate with similar transcription factor strength of ToxR. This is particularly evident in the ToxR^C293S^ mutant, which displayed a higher transcription activity than the ToxR^C236S^ mutant or ToxR^WT^. Apart from steric limitations, the cysteine residues in ToxR may be able to assemble into tertiary or quaternary structures. For example, monomeric ToxR could be found in a reduced or oxidized state by the formation of intramolecular disulfide bonds. Furthermore, ToxR could build intermolecular disulfide bonds by a C236‐C236S, C293‐C293 or C236‐C293 linkage or oligomers, which would be connected in a chain‐like conformation.

Cysteine‐dependent homodimerization seemingly provokes ToxR activation, but equally important is the formation of intramolecular disulfide bonds, which are needed to stabilize ToxR molecules (Lembke *et al.*, 2018). Although disulfide bonds are covalent linkages, they can be isomerized enzymatically by the correction system DsbCD (Figure 7). If ToxR, once activated and locked by intermolecular disulfide bonds, cannot be deactivated anymore, it may not be able to respond to changing environmental signals. This would cause severe problems in the stress response or energy homeostasis of the cell. Therefore, we examined the process of intra‐ and intermolecular disulfide bond formation in ToxR and were able to demonstrate that the Dsb system in the periplasm introduces and controls the correct arrangement of disulfide bonds in ToxR. We were able to confirm that DsbA is the primary electron donor for ToxR and ToxR^C293S^ cysteine linkages (Lembke *et al.*, 2018), assuming that their cysteine residues are in very close proximity (Landeta *et al.*, 2018). DsbA possesses one of the highest redox potential values (−120 mV) among many known thiol‐disulfide oxidoreductases. This leads to rapid disulfide bond pairings, but these do not necessarily occur between the correct combinations of cysteines (Wunderlich *et al.*, 1975; Grauschopf *et al.*, 1995). Therefore, DsbA and DsbB must cooperate with the disulfide bond isomerization system DsbC and DsbD, to achieve the native proteome through the correction of false disulfide bonds (Kadokura *et al.*, 2003). We found that DsbC is responsible for the formation of intermolecular disulfide bonds in ToxR homodimers. Furthermore, the lack of DsbA or DsbC, affected the regulation potency of native ToxR, best observed for *ompU* expression. This observation is in line with the decreased homodimer formation evidenced in overexpression studies. Unfortunately, we were unable to detect homodimers from native chromosomal expressed *toxR*. Nonetheless, we propose the following scenario (Figure 7). During de novo protein biosynthesis, the insertion of ToxR into the membrane exposes its thiol groups in the periplasm, which are, in turn, oxidized by the DsbAB system to form intramolecular disulfide bonds. DsbC then catalyses the exchange of ToxR disulfide bonds formed by DsbA under conditions of dimer formation that favor ToxR transcriptional activity and temporary homodimer conformations. It is tempting to speculate that ToxR homodimers are only transiently linked by an intermolecular disulfide bond under activating conditions, thus native ToxR homodimers may not be the abundant forms and therefore hard to detect. However, as soon as *V. cholerae* experiences less‐activating conditions, DsbC would conceivably dissolve this cysteine bridge, and ToxR would switch back to its monomeric form stabilized by intramolecular disulfide linkages. Further studies will be carried out to decipher the mechanisms of this redox switch.

Cysteines are important to the structure of proteins—they provide proteins with greater stability and allow them to better respond to environmental cues. However, cysteines can also cause incorrect folding. The operon partner of ToxR, ToxS, appears to stabilize ToxR in a conformation that is optimized for transcriptional activation (DiRita and Mekalanos, 1991; Ottemann and Mekalanos, 1996; Dziejman *et al.*, 1999; Midgett *et al.*, 2017; Lembke *et al.*, 2018). Our results confirm the observations by Midgett *et al.* by showing ToxR–ToxS physical interactions using BACTH in *E. coli* (Midgett *et al.*, 2017). We show that the interaction of ToxR with ToxS was significantly increased compared to ToxR–ToxR interactions in the absence of ToxS. Furthermore, we show that ToxS significantly increases ToxR–ToxR PPIs probably through the interaction of ToxR with ToxS itself. This was independent of the DNA‐binding capacity of ToxR, as demonstrated using a DNA‐binding‐deficient ToxR^W76R^ mutant. Noteworthy, when ToxR is able to bind to its ToxR boxes (e.g., *V. cholerae ompU* operators) and ToxS is present, the maximum ToxR‐ToxR PPI was observed. To mention, disulfide‐linked homodimer formations decreased when *toxS* was co‐expressed in *V. cholerae*. However, our BACTH data in *E. coli* showed that ToxS concurrently enhances ToxR‐ToxR PPIs. It may be speculated that ToxS mitigates ToxR homodimer formation to diminish premature disulfide bond formations and favor specific ToxR interactions that may convert into an optimized transcriptional active complex that exhibits an ideal conformation for operator binding (Figure 7). Furthermore, ToxS may keep inter‐molecular disulfide bonds labile and therefore counteracted the DsbC isomerase action, which ultimately leads to the switching back and forth between inter‐ and intramolecular disulfide bonds. However, we need to interpret such data carefully, since overexpression of proteins may cause artificial effects. Further studies are needed to evaluate this issue.

Finally, we unraveled that DC was able to further enhance ToxR‐ToxR PPIs when *toxS* was co‐expressed. These observations extend the results published by Midgett *et al.*, who reported that chenodeoxycholate interacts with the purified periplasmic domain of ToxR which then leads to enhanced interactions between ToxR and ToxS (Midgett *et al.*, 2017). This indicates that DC may facilitate ToxR cooperativity to further stabilize or support the interactions within the ToxRS complex (Figure 7). To decipher this mechanism, future studies are needed to solve the protein structure of the ToxRS complex co‐crystallized with DC to identify possible conformational (e.g., homo‐, heterodimerization or sub‐domain) changes.

In this report, we focused on the molecular mechanism of the ToxR activation process mainly restricted to the *ompU* and *ompT* promoters, which are known to respond to bile. Subsequently, OmpU then confers the bacteria to bile resistance, an important physiological adaptation process, during the course of colonization in humans (Provenzano *et al.*, 2000; Provenzano and Klose, 2000). Our obtained results are in accordance with previous observations that show that dimerization and other PPIs occur between the ToxR and ToxR‐ToxS molecules. This study extends the current view by showing that such ToxR PPIs are dynamic in response to ToxR‐boxes, cysteine disulfide bond formations, ToxS and the presence of bile (DC) (Figure 7). Since the binding ability for *ompU*‐operators increases ToxR PPIs in the presence of ToxS and the periplasmic domain plays a major role here, shown by the *toxR^C293S^* mutant, a signal path that leads from the inside to the outside seems very likely for ToxR. Still, it would be intriguing to speculate that environmental factors would initiate ToxR PPIs in an outside‐to‐inside direction. Here, the first hint is derived from bile (DC), representing an extracellular signal molecule. We show that it enhances the PPIs of ToxR in a similar manner in the presence of ToxS. In light of our results, we propose that sequential activation requirements such as that of ToxR may initially only form labile ToxR‐ToxR contacts. Such a preliminary complex then associates and gets stabilized by ToxS. If bile is present, more tightly bound ToxRS complexes are formed. Their binding to DNA operator binding sites then increases ToxR‐ToxR PPIs to the maximum. We hypothesize that the stability of ToxR complexes is further increased by the formation of transient intermolecular disulfide bonds during DNA binding, which is catalyzed by DsbA/DsbC, while PPIs between ToxR and ToxS may be reduced (Figure 7). Most of our data were derived from in vitro experiments; further in vivo studies will be carried out to determine the biological relevance of our findings. Regarding the mechanism of ToxR dimerization, some interesting questions still remain to be answered: what is the strength of the interaction between ToxR molecules and how does this change during the interplay with environmental factors; when exactly do ToxS‐ToxR complexes arise and when do they dissolve; does the binding of ToxR to its DNA operator sites result in conformational changes? Further comprehensive analyses of the mechanisms of action are needed to clarify these questions.

## 4 EXPERIMENTAL PROCEDURES

### 4.1 Strains, plasmids, and culture conditions

All bacterial strains and plasmids used in this study are listed in Table 1. Here, *V. cholerae* O1 El Tor Inaba P27459‐S was used as the wild‐type (WT) strain (Pearson *et al.*, 1993). The *E. coli* strains XL1‐Blue, DH5α λpir, BL21 (DE3), and SM10 λpir were used for cloning, plasmid propagation, and conjugation (Kolter *et al.*, 1978; Hanahan, 1983; Miller and Mekalanos, 1988), (New England Biolabs). Unless indicated otherwise, bacteria were routinely grown with aeration in lysogeny broth (LB), M9 maltose minimal medium or MacConkey maltose agar plates or in the respective liquid medium at 180 rpm at 37°C. When appropriate, supplements were added at the following final concentrations: streptomycin (Sm; 100 μg/ml), ampicillin (Ap; 50 or 100 μg/ml), chloramphenicol (Cm; 2 μg/ml), kanamycin (Km; 50 μg/ml), l‐arabinose (0.1%), sucrose (10%), maltose (0.2% or 1%), glucose (0.2%), isopropyl ß‐d‐1‐thiogalactopyranoside (IPTG; 0.05 or 0.5 mM), and sodium‐deoxycholate (DC; 0.1%).

**TABLE 1:** Strains and plasmids used in this study

Strains/Plasmids	Descriptions	References
*E. coli* strains		
DH5αλpir	*F^‐^* Δ(*lacZYA‐argF*)*U169 recA1 endA1 hsdR17 supE44 thi‐1 gyrA96 relA1* λ::*pir*	Hanahan, (1983)
SM10λpir	*thi thr leu tonA lacY supE recA*::RPA‐2‐Te::Mu λpirR6K, Km^r^	Miller and Mekalanos (1988)
XL1‐Blue	*F* ^‐^ *::Tn10 proA^+^B^+^ lac^q^* Δ*(lacZ)M151 recA1 endA1 gyrA46 (Nal^r^) thi hsdR17 (r_K_^−^m_K_^+^) supE44 relA1 lac*	Bullock *et al. *(1987)
BL21 (DE3)	*fhuA2 [lon] ompTgal (λ DE3) [dcm] ∆hsdS λ DE3=λsBamHIo ∆EcoRI‐B int::(lacI::PlacUV5::T7 gene1) i21 ∆nin5*	NEB
W3110 Δ*cyaA*	F^‐^ λ^‐^ *rpoS*(*Am*) *rph‐1 Inv*(*rrnD*‐*rrnE*) Δ*cyaA*::*scar*	Herbst *et al. *(2018)
*V. cholerae* strains		
WT	P27459‐S, O1 Inaba, El Tor, clinical isolate, Bangladesh 1976, spontaneous Sm^r^	Pearson *et al. *(1993)
Δ*toxR*	P27459‐S with deletion in *toxR*, Sm^r^	Fengler *et al. *(2012)
Δ*toxRS*	P27459‐S with deletion in *toxR* and *toxS*, Sm^r^	Fengler *et al. *(2012)
*toxR^CC^*	P27459‐S with *toxR* replaced by FLAG‐*toxR^C236SC293S^,* Sm^r^	Fengler *et al. *(2012)
*toxR^C236S^*	P27459‐S with *toxR* replaced by FLAG‐*toxR^C236S^,* Sm^r^	This study
*toxR^C293S^*	P27459‐S with *toxR* replaced by FLAG‐*toxR^C293S^,* Sm^r^	This study
*toxR^C293W76RS^*	P27459‐S with *toxR* replaced by FLAG‐*toxR^C293SW76R^,* Sm^r^	This study
Δ*dsbA*	P27459‐S with *dsbA* replaced by *km* cassette, Sm^r^, Km^r^	Fengler *et al. *(2012)
*dsbC*::pGP	P27459‐with *dsbC* inserted by pGP704, Sm^r^, Ap^r^	Fengler *et al., *(2012)
Δ*dsbA toxR^C293S^*	P27459‐S Δ*dsbA* with *toxR* replaced by FLAG‐*toxR^C293S^*, Sm^r^ *,* Km^r^	This study
*dsbC*::pGP *toxR^C293S^*	P27459‐S *dsbC*::pGP with *toxR* replaced by FLAG‐*toxR^C293S^*, Sm^r^ *,* Ap^r^	This study
Δ*degP*	P27459‐S with *degP* replaced by *cat* cassette, Sm^r^, Cm^r^	This study
Δ*degP ompU*::*phoA*	P27459‐S Δ*degP* with insertion of pGP704phoA downstream of *ompU*, Sm^r^ *,* Cm^r^, Ap^r^	This study
Δ*toxR ompU*::*phoA*	P27459‐S with deletion in *toxR* and insertion of pGP704phoA downstream of *ompU*, Sm^r^ *,* Cm^r^, Ap^r^	This study
Δ*degP toxR^CC^ ompU*::*phoA*	P27459‐S Δ*degP* with *toxR* replaced by FLAG‐*toxR^C236SC293S^* and insertion of pGP704phoA downstream of *ompU*, Sm^r^, Cm^r^, Ap^r^	This study
Δ*degP toxR^C236S^ ompU*::*phoA*	P27459‐S Δ*degP* with *toxR* replaced by FLAG‐*toxR^C236S^* and insertion of pGP704phoA downstream of *ompU*, Sm^r^ *,* Cm^r^, Ap^r^	This study
Δ*degP toxR^C293S^ ompU*::*phoA*	P27459‐S Δ*degP* with *toxR* replaced by FLAG‐*toxR^C293S^* and insertion of pGP704phoA downstream of *ompU*, Sm^r^ *,* Cm^r^, Ap^r^	This study
Δ*degP ompT*::*phoA*	P27459‐S Δ*degP* with insertion of pGP704phoA downstream of *ompT*, Sm^r^ *,* Cm^r^, Ap^r^	This study
Δ*toxR ompT*::*phoA*	P27459‐S with deletion in *toxR* and insertion of pGP704phoA downstream of *ompT*, Sm^r^ *,* Cm^r^, Ap^r^	This study
Δ*degP toxR^CC^ ompT*::*phoA*	P27459‐S Δ*degP* with *toxR* replaced by FLAG‐*toxR^C236SC293S^* and insertion of pGP704phoA downstream of *ompT*, Sm^r^ *,* Cm^r^, Ap^r^	This study
Δ*degP toxR^C236S^ ompT*::*phoA*	P27459‐S Δ*degP* with *toxR* replaced by FLAG‐*toxR^C236S^* and insertion of pGP704phoA downstream of *ompT*, Sm^r^ *,* Cm^r^, Ap^r^	This study
Δ*degP toxR^C293S^ ompT*::*phoA*	P27459‐S Δ*degP* with *toxR* replaced by FLAG‐*toxR^C293S^* and insertion of pGP704phoA downstream of *ompT*, Sm^r^ *,* Cm^r^, Ap^r^	This study
Plasmids		
pKEK229	Ori_R6K_, *mobRP4*, *sacB*, Ap^r^	Correa *et al. *(2000)
pCVD442	Ori_R6K_, *mobRP4*, *sacB*, Ap^r^	Donnenberg and Kaper (1991)
pGP704	Ori_R6K_, *mobRP4*, Ap^r^	Miller and Mekalanos (1988)
pBAD18‐Kan	Expression vector, ori_ColE1_, arabinose Inducible, Km^r^	Guzman *et al. *(1995)
pBAD18	Expression vector, ori_ColE1_, arabinose Inducible, Ap^r^	Guzman *et al. *(1995)
pACYC184	Cloning vector, orip15A, Tet^r^, Cm^r^	Rose (1988)
pFLAG‐MAC^TM^	Expression vector with N‐terminal FLAG‐Tag, IPTG inducible, Ap^r^	Sigma‐Aldrich
pKT25	Expression vector, encodes for the first 224 AA of CyaA (T25 fragment, *B. pertussis*), C‐terminal heterologous protein fusion, IPTG inducible, Km^r^	Karimova *et al. *(1998)
pUT18C	Expression vector, encodes for AA 225 to 399 of CyaA (T18 fragment, *B. pertussis*), C‐terminal heterologous protein fusion, IPTG inducible, Ap^r^	Karimova *et al. *(1998)
pKEK229dsbA::km	pKEK229 carrying up and down fragments, Ap^r^, Km^r^	Fengler *et al. *(2012)
pCVD442degP::cat	pCVD442 carrying up and down fragment of *degP* flanking a *cat* cassette, Ap^r^, Cm^r^	Vorkapic *et al. *(2019)
pCVD442toxR	pCVD442 carrying up and down fragment of *toxR*, Ap^r^	Fengler *et al. *(2012)
pCVD442FLAGtoxR^CC^	pCVD442 carrying up and down fragment of FLAGtoxR^C236C293S^, Ap^r^	Fengler *et al. *(2012)
pCVD442FLAGtoxR^C236S^	pCVD442 carrying up and down fragment of FLAGtoxR^C236S^, Ap^r^	This study
pCVD442FLAGtoxR^C293S^	pCVD442 carrying up and down fragment of FLAGtoxR^C293S^, Ap^r^	This study
pCVD442FLAGtoxR^C293SW76R^	pCVD442 carrying up and down fragment of FLAGtoxR^C293SW76R^, Ap^r^	This study
pFLAGtoxR	*toxR* of P27495‐S in pFLAG‐MAC^TM^, Ap^r^	Fengler *et al. *(2012)
pFLAGtoxRS	*toxR* and *toxS* of P27495‐S in pFLAG‐MAC^TM^, Ap^r^	Fengler *et al. *(2012)
pFLAGtoxR^CC^	*toxR^C236SC293S^* point mutant of P27495‐S in pFLAG‐MAC^TM^, Ap^r^	Fengler *et al. *(2012)
pFLAGtoxR^CC^toxS	*toxR^C236SC293S^* point mutant and *toxS* of P27495‐S in pFLAG‐MAC^TM^, Ap^r^	Fengler *et al. *(2012)
pFLAGtoxR^C236S^	*toxR^C236S^* point mutant of P27495‐S in pFLAG‐MAC^TM^, Ap^r^	This study
pFLAGtoxR^C293S^	*toxR^C293S^* point mutant of P27495‐S in pFLAG‐MAC^TM^, Ap^r^	This study
pFLAGtoxR^W76R^S	*toxR^W76R^S* of point mutant of P27495‐S in pFLAG‐MAC^TM^, Ap^r^	This study
pBAD18‐KanFLAGtoxRS	FLAG‐*toxRS* of P27495‐S in pBAD18‐Kan, Km^r^	This study
pBAD18‐FLAGtoxRS	FLAG‐*toxRS* of P27495‐S in pBAD18, Ap^r^	This study
pGP704dsbC	pGP704 carrying internal fragment of *dsbC,* Ap^r^	Fengler *et al. *(2012)
pGP704phoAompU	pGP704phoA with *ompU* gene fragment, Ap^r^	Lembke *et al. *(2018)
pGP704phoAompT	pGP704phoA with *ompT* gene fragment, Ap^r^	Lembke *et al. *(2018)
pKT25‐zip	BACTH positive control, leucine zipper of GCN4 (yeast) fused to the T25 fragment, Km^r^	Karimova *et al. *(1998)
pUT18C‐zip	BACTH positive control, leucine zipper of GCN4 (yeast) fused to the T18 fragment, Ap^r^	Karimova *et al. *(1998)
pKT25toxR	*toxR* of P27495‐S in pKT25, Km^r^	This study
pKT25toxRS	*toxR* and *toxS* of P27495‐S in pKT25, Km^r^	This study
pKT25toxR^W76R^	*toxR^W76R^* of P27495‐S in pKT25, Km^r^	This study
pKT25toxR^W76R^S	*toxR^W76R^* and *toxS* of P27495‐S in pKT25, Km^r^	This study
pUT18CtoxR	*toxR* of P27495‐S in pUT18C, Ap^r^	This study
pUT18CtoxRS	*toxR* and *toxS* of P27495‐S in pUT18C, Ap^r^	This study
pUT18CtoxR^W76R^	*toxR^W76R^* of P27495‐S in pUT18C, Ap^r^	This study
pUT18CtoxR^W76R^S	*toxR^W76R^* and *toxS* of P27495‐S in pUT18C, Ap^r^	This study
pUT18CtoxSFLAG	*toxS*‐FLAG of P27495‐S in pUT18C, Ap^r^	This study
pUT18Cop^ompU^	*ompU* operators O123 of P27495‐S in pUT18C, Ap^r^	This study
pUT18Cop^ompU^ toxR	*toxR* of P27495‐S in pUT18Cop^ompU^, Ap^r^	This study
pUT18Cop^ompU^ toxRS	*toxR* and *toxS* of P27495‐S in pUT18Cop^ompU^, Ap^r^	This study
pUT18Cop^ompU^ toxR^W76R^	*toxR^W76R^* of P27495‐S in pUT18Cop^ompU^, Ap^r^	This study
pUT18Cop^ompU^ toxR^W76R^S	*toxR^W76R^* and *toxS* of P27495‐S in pUT18Cop^ompU^, Ap^r^	This study

### 4.2 Strain and plasmid constructions

The primer (Thermo Fisher Scientific) used in this study for amplification as well as sequencing are listed in Table 2. PCR products and vectors were digested with the respective restriction endonucleases, ligated with T4 DNA ligase (NEB) and sequenced for validation (LGC Genomics) (data not shown).

**TABLE 2:** Oligonucleotides^a^ (5ʹ‐3ʹ) used in this study

Oligonucleotides (5′‐3′) used in this study	
BACTH	
Pst_pKT25_ToxR_fwd_BACTH	ATTCTGCAGTCGGATTAGGACACAACTC
Pst_pUT18C_ToxR_fwd_BACTH	ATTCTGCAGTTTCGGATTAGGACACAACT
XbaI_ToxR_rev_BACTH	ATTTCTAGACTACTCACACACTTTGATGG
ToxS_XbaI‐fwd	TAATCTAGAGATGCAAAATAGACACATCGCC
ToxS‐FLAG_EcoRI‐rev	TATGAATTCTGAAAATCTTCTCTCACTCGA
XbaI_ToxS_rev	ATTTCTAGATTAAGAATTACTGAACAGTACG
BamHI_ompUO123_fwd	ATTGGATCCTCCTAAATCGGGTCGGGT
KpnI_ompUO123_rev	AAAGGTACCATTGGTCATTGTTGTGTTCA
*toxR^C236S^* and *toxR^C293S^* substitution	
HindIII_toxR_5ʹ_FLAG	AATAAGCTTATGTTCGGATTAGGACACAACTCA
KpnI_toxR_3ʹ_FLAG	AATGGTACCCTACTCACACACTTTGATGGCAT
KpnI_toxR293S_3ʹ_FLAG	AATGGTACCCTACTC**AGA**CACTTTGATGGCATCGTTA
toxRC236S_5ʹ	GGCTACCGTCAATCGAAC**TGA**GCGTTAAAAAATACAATGA
toxRC236S_3ʹ	TCATTGTATTTTTTAACGC**TCA**GTTCGATTGACGGTAGCC
SacI_toxRS_1	TTTGAGCTCATTTGGAAATCACATCGCGCAAAC
XbaI_toxRS_4	TTTTCTAGAATGACGTTTCCCCGCGGTGAG
c_FLAGtoxR_3ʹ_F1	TGTCATCGTCGTCCTTGTAGTCCATCTAATGTCCCAGTATCTCCCTGT
c_FLAGtoxR_5ʹ_F2	GGGACAGGGAGATACTGGGACATTAGATGGACTACAAGGACGACGATGA
c_FLAGtoxR_3ʹ_F2	CTACTCACACACTTTGATGGCAT
c_FLAGtoxRC293S_3ʹ_F2	CTACTC**AGA**CACTTTGATGGCAT
c_FLAGtoxR_5ʹ_F3	AACCAGTTAACGCTGAATTACATTC
c_FLAGtoxRC293S_5ʹ_F3	GTTGCTAACCCTAACGATGCCATCAAAGTG**TCT**GAG
*toxR^C293SW76R^* substitution	
F1_ToxRC293S‐W76R_XbaI_fwd	TTATCTAGAATCCGCCACGATGAAAGCCGA
F1_SOE_ToxRC293S‐W76R_rev	TTGCTCTCG**CCG**AACAAAGTCATGCAAATCATTGCGAGA
F2_SOE_ToxRC293S‐W76R_fwd	GACTTTGTT**CGG**CGAGAGCAAGGTTTTGAAGTCGATGAT
F2_ToxRC293S‐W76R_SacI_rev	TAAGAGCTCCAGACCGCAGCATCCAATTGC
pBAD18‐KanFLAGtoxRS, pBAD18‐FLAGtoxRS, pFLAGtoxR^W76R^S	
fwd_SacI_pFlagMAC_ShineD	TTAGAGCTCATAACAATTTCACACAGGAGA
FLAGtoxR_fw_KpnI	ATAGGTACCATGTTCGGATTAGGACACAACTCA
BglII_toxRS_3ʹFLAG	TTAAGATCTTTAAGAATTACTGAACAGTACGGT
Sequencing	
phoA‐seq‐rev	GCTCACCAACTGATAACCAC
SacIDsbA1	TTTGAGCTCCAAGAAGAGATCCCGATCGTC
Kan_cassette_rv	TTAGAAAAACTCATCGAGCA
PhoA3ʹ 180 rev	GCTAAGAGAATCACGCAGA
pGP704_CVD_rv|15	GATGTAACGCACTGAGAAG
pBAD_fwd	CCATAGCATTTTTATCCATAAG

Restriction sites are underlined. Bold letters indicate codons changed to obtain desired amino acid mutations.

Suicide plasmids generating chromosomal deletions, amino acid substitutions or *phoA* fusions were achieved via PCR or SOE‐PCR (splicing by overlap extension) (Horton *et al.*, 1989). Creation of deletion mutants was performed by cloning two DNA fragments of approximately 800 bp representing upstream and downstream of the target gene into pCVD442 (Donnenberg and Kaper, 1991). ToxR amino acid substitutions C236S and C293S were constructed using c_FLAGtoxR_5ʹ_F2, c_FLAGtoxR_3ʹ_F2, c_FLAGtoxRC293S_3ʹ_F2, SacI_toxRS_1, c_FLAGtoxR_3ʹ_F1, XbaI_toxRS_4, c_FLAGtoxR_5ʹ_F3 and c_FLAGtoxRC293S_5ʹ_F3, respectively. Fragments were amplified from pFLAGtoxR^C236S^, pFLAGtoxR^C293S^ or chromosomal WT DNA to create pCVD442FLAGtoxR^C236S^ and pCVD442FLAGtoxR^C293S^, respectively. Chromosomal DNA of *V. cholerae* P27459‐S Δ*toxR*::FLAG*toxR^C293S^* served as a template to generate the suicide plasmid pCVD442FLAGtoxR^C236SW76R^. The W76R point mutation (Morgan *et al.*, 2011) in *toxR* was generated by SOE PCR utilizing primers listed in Table 2, subitem *toxR^C293SW76R^* substitution. The resulting plasmids were isolated from *E. coli* DH5α λpir, transformed into SM10 λpir and subsequently introduced into *V. cholerae* derivatives by conjugation (Donnenberg and Kaper, 1991). Transconjugants were selected on LB plates containing streptomycin and ampicillin. Sucrose counter‐selection and further selection steps were performed as described previously (Donnenberg and Kaper, 1991).

For the construction of the expression plasmid pFLAGtoxR^C293S^ template DNA of the WT was used together with the primer pair HindIII_toxR_5ʹ_FLAG and KpnI_toxR293S_3ʹ_FLAG with the latter containing a point mutation within the DNA sequence that changed Cys293 to Ser293. The C236S point mutation in pFLAGtoxR^C236S^ was generated by SOE PCR, using pFLAGtoxR as a template together with primers HindIII_toxR_5ʹ_FLAG, toxRC236S_3ʹ, toxRC236S_5ʹ, and KpnI_toxR_3ʹ_FLAG. Primers fwd_SacI_pFlagMAC_ShineD and XbaI_ToxS_rev were used to amplify PCR fragments derived from pFLAGtoxRS to construct pBAD18‐KanFLAGtoxRS and pBAD18FLAGtoxRS. The *toxR*, *toxRS*, *toxR^W76R^*, *toxR^W76R^S*, *toxS*‐FLAG, and *ompU* O123 operator fragments in pKT25 or pUT18C for the BACTH system were amplified by PCR using the BACTH primers listed in Table 2. The coding regions originate from WT DNA or pFLAGtoxR^W76R^S plasmid DNA which itself was generated by SOE PCR using primers FLAGtoxR_fw_KpnI, BglII_toxRS_3ʹFLAG, F1_SOE_ToxRC293S‐W76R_rev, and F2_SOE_ToxRC293S‐W76R_fwd. Subsequently, the pFLAG‐MAC^TM^, pKT25, pUT18C (IPTG inducible) and pBAD (arabinose inducible) plasmids were electroporated into DH5α λpir, XL1‐Blue or BL21 (DE3) and monitored for expression before being introduced into *E. coli* W3110 Δ*cyaA* or *V. cholerae* derivatives.

### 4.3 Generation of cell extracts and immunoblot analysis

To verify protein expression in *V. cholerae* and *E. coli*, immunoblotting was performed. Whole cell lysates (WCL) were taken from cultures grown in LB overnight which were used to inoculate fresh LB or M9 maltose minimal medium to an OD_600_ of 0.1. Cells were grown at 37°C and 180 rpm until the mid‐log phase (OD_600_ = 0.4–0.6) was reached and subsequently collected before or after induction with IPTG (0.05–0.5 mM) or arabinose (0.1%) for 2 hr. Cells were resuspended in Laemmli buffer (Laemmli, 1970) with or without β‐mercaptoethanol, corresponding to reducing and non‐reducing conditions, respectively. The overall protein contents were analyzed to contain similar protein levels as described previously (Lembke *et al.*, 2018). Following transfer on a AmershamTM ProtranTM 0.45‐µm nitrocellulose membrane (GE Healthcare Life Sciences), the membranes were blotted for OmpU, OmpT, ToxR or FLAG‐tagged proteins respectively (mouse anti‐OmpU and anti‐OmpT 1:3,000 (Salem *et al.*, 2015), rabbit anti‐ToxR 1:1,000 (Fan *et al.*, 2014), mouse anti‐FLAG M2 Peroxidase (HRP) 1:2,000 (Sigma)). The washing steps were performed as described previously (Lembke *et al.*, 2018). Peroxidase secondary antibodies (horseradish peroxidase‐conjugated goat anti‐rabbit 1:10,000 or goat anti‐mouse 1:7,500 Dianova GmbH) were used for detection using ECL solution (Clarity™ Western ECL Blotting Substrates, BIO‐RAD) prior to visualization of the reactive protein bands using a Molecular Imager ChemiDocTM XRS System (BIO‐RAD). Quantification of ToxR protein band intensities was performed using Image Lab Software (BIO‐RAD). One immunoblot used for this analysis is shown (Figure S5).

### 4.4 Bacterial two‐hybrid analysis (BACTH)

For monitoring of protein–protein interactions in vivo, the bacterial adenylate cyclase‐based two‐hybrid (BACTH) system was performed as described in Karimova *et al*. (1998). *V. cholerae* ToxR and ToxRS derivatives or ToxS‐FLAG were fused to the 3ʹ end of the adenylate cyclase T25 or T18 fragment from *Bordetella pertussis* (CyaA), respectively. Additionally, *V. cholerae ompU* operators (op*^ompU^*) were cloned on pUT18C. Prior to the interaction studies, plasmid functionality was tested in *E. coli* XL1‐Blue, Dh5αλpir or BL21 (DE3) by expression and sequencing. Oligonucleotide primers for cloning onto pKT25 and pUT18C plasmids are listed in Table 2. Positive complementary pUT18CtoxSFLAG, pKT25toxR and pUT18CtoxR plasmids (and its derivatives) were co‐transformed in *E*. *coli* K‐12 strain W3110 ∆*cyaA* (Herbst *et al.*, 2018), which lacks endogenous adenylate cyclase activity. The transformants were selected on MacConkey agar (Becton Dickinson) plates supplemented with 50 µg/ml of ampicillin, 50 µg/ml of kanamycin, and 1% maltose for 24 hr at 30°C to reveal the CyaA^+^ phenotype (red colonies indicate maltose fermentation). For a clear presentation of PPIs, cells were grown at 37°C in LB medium overnight using selection for kanamycin and ampicillin. Subsequently, 5 µl of overnight culture was transferred directly onto a single MacConkey indicator plate to be compared and incubated for 24 hr at 30°C. As a positive complementation control, the leucine zipper of the yeast GCN4 protein was used (pKT25‐zip and pUT18C‐zip) whereas the empty pKT25 and pUT18C plasmids served as negative controls.

### 4.5 β‐Galactosidase and alkaline phosphatase assays

To determine transcriptional activity of chromosomal ToxR, pGP704phoAompU and pGP704phoAompT were introduced into *V. cholerae* derivatives by conjugation. Strains were inoculated from selective LB overnight cultures to an OD_600_ of 0.1 and grown in fresh selective M9 maltose minimal medium at 37°C and 180 rpm until the mid‐log phase (OD_600_ = 0.4–0.6) was reached. For the quantitative analysis of ToxR‐ToxR or ToxR‐ToxS‐FLAG protein–protein interactions in vivo, the BACTH system was used in *E. coli* W3110 ∆*cyaA*. The method is based on the positive regulation of β‐galactosidase expression by cAMP/CAP that will be produced upon functional complementation of the chimeric adenylate cyclase. There cultures were inoculated from selective LB overnight cultures to an OD_600_ of 0.1 and grown in fresh selective LB medium supplemented with 0.05 mM IPTG at 37°C and 180 rpm to the stationary phase in the absence or presence of 0.1% DC. For each assay 1–2 ml of culture was harvested by centrifugation respectively. Enzymatic activities for LacZ and PhoA were measured as described previously (Taylor *et al.*, 1987; Miller, 1992) with at least three biological replicates each with technical triplicates.

### 4.6 Statistical analysis

The statistics in the respective experiments were carried out using GraphPad Prism 6.

## Supporting information

Supplementary Material
