# Supplementary material for: Host stimuli and operator binding sites controlling protein interactions between virulence master regulator ToxR and ToxS in Vibrio cholerae
Source: Mol Microbiol. 2020 Apr 19;114(2):262–78. doi: 10.1111/mmi.14510 (PMC7496328; doi:10.1111/mmi.14510)
Supplement: Supplementary file 1 — Supplementary Material [file MMI-114-262-s001.pdf]

Supplementary Information

Host stimuli and operator binding sites controlling protein interactions between virulence master regulator ToxR and ToxS in *Vibrio cholerae*

Authors:

Mareike Lembke[a], Thomas Höfler[a], Ada-Natsuko Walter[a], Sarah Tutz[a], Vera Fengler[b], Stefan Schild[a,c,d], and Joachim Reidl[a,c,,d #]

[a]Institute of Molecular Biosciences, University of Graz, Humboldtstraße 50, 8010 Graz, Austria

[b]Division of Physiological Chemistry, Medical University of Graz, Neue Stiftingtalstraße 6, 8010 Graz, Austria

[c]BioTechMed-Graz, A-8010 Graz, Austria

[d]BioHealth, University of Graz, A-8010, Austria

[#]Corresponding author mailing address: Institute of Molecular Biosciences, University of Graz, Humboldtstraße 50, A-8010 Graz, Austria. Phone: +43 316 380 1978. Fax: +43 316 380 9019. E-mail: joachim.reidl@uni-graz.at

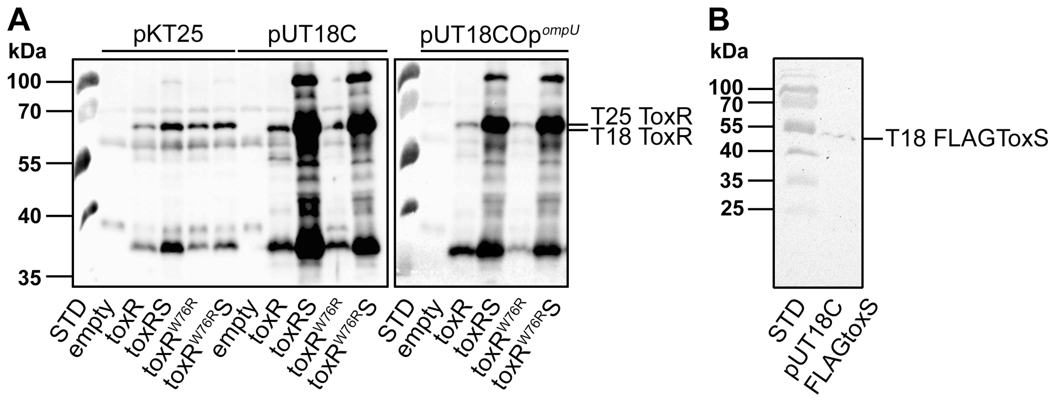

Figure S1 Expression of single BACTH plasmids in *E. coli* Dh5αλpir, XL1-Blue and BL21 (DE3). *E. coli* strains carrying *toxR* derivatives with or without *toxS* or *toxS*-FLAG alone on pKT25, pUT18C or pUT18COp^{ompU} were grown in LB. Samples were harvested after 2 h induction with 0.5 mM IPTG in the mid-log phase and analyzed by immunoblotting using anti-ToxR (**A**) or anti-FLAG (**B**) antibodies, respectively. Immunoblots were performed under standard reducing Laemmli buffer conditions. Standard (STD): PageRuler Prestained Protein Ladder (10 to 180 kDa) (Thermo Fisher Scientific).

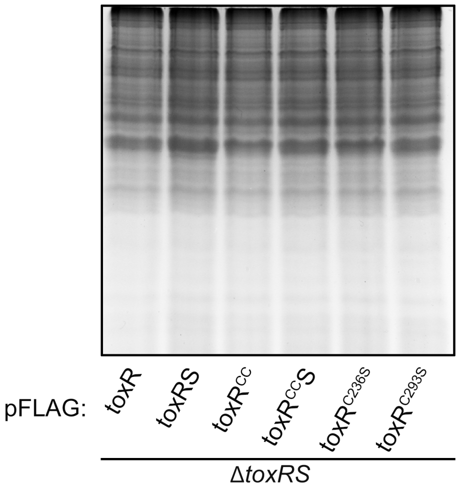

Figure S2 Protein amounts of overexpressed ToxR and derivatives. WCL of *V. cholerae* Δ*toxRS* strains carrying *toxR* derivatives with or without its operon partner *toxS* on pFLAG-MAC™ were analyzed by SDS-PAGE under standard non-reducing Laemmli buffer conditions and Kang staining (Kang *et al.*, 2002) as loading controls for the immunoblot displayed in Fig. 3. Cells were grown in LB and samples were taken after 2 h induction with 0.05 mM IPTG in the mid-log phase.

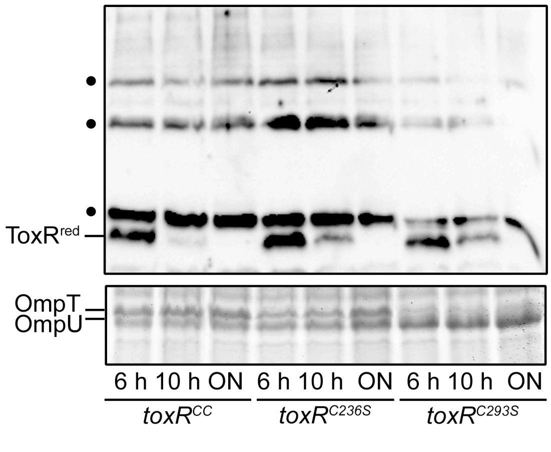

Figure S3 Protein amounts and transcription factor activity of *toxR* cysteine mutants. *V. cholerae toxR* cysteine mutants were grown in M9 maltose minimal medium and samples were taken at the time indicated. WCL were subjected to immunoblot and probed with anti-ToxR antibodies to detect ToxR protein levels under standard reducing conditions. Simultaneously, the protein content was analyzed for OmpU and OmpT expression by SDS-PAGE and Kang staining (Kang *et al.*, 2002). (•) Represents nonspecific cross-reacting background bands.

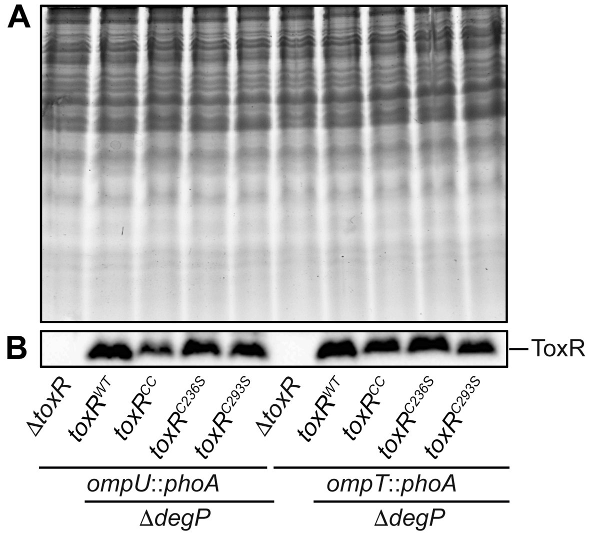

Figure S4 ToxR expression in strains used for OmpU/T profile studies *V. cholerae* Δ*degP toxR* cysteine mutants harbouring pGP704phoAompU or pGP704phoAompU insertions (see Fig. 4) were grown in M9 maltose minimal medium and samples were taken in the mid-log phase. WCL were analyzed under standard reducing Laemmli buffer conditions by SDS-PAGE in combination with **A** Kang-staining as loading controls (Kang *et al.*, 2002) or **B** immunoblot analysis utilizing anti-ToxR antibodies.

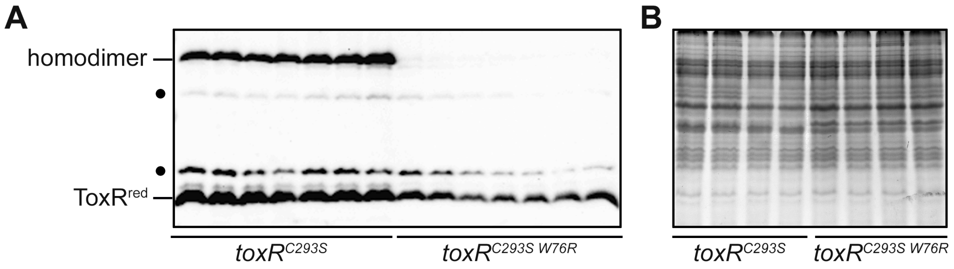

Figure S5 DNA binding triggers ToxR homodimer formation. *V. cholerae toxR*^{C293S} and *toxR*^{C293SW76R} cells were grown in M9 maltose minimal medium until the mid-log phase was reached. **A** Representative immunoblot for densitometric analysis of ToxR homodimer and monomer formation in *V. cholerae toxR*^{C293S} and *toxR*^{C293SW76R} strains displayed in Figure 5 B. WCLs of four biological replicates each with a technical replicate were loaded on one SDS gel. Immunoblotting was performed under standard non-reducing Laemmli buffer conditions using anti-ToxR antibodies. ToxR band intensities (%) were readout using Image lab software (BIO-RAD). (•) Represents nonspecific cross-reacting background bands. **B** WCL loading control of four biological *V. cholerae toxR*^{C293S} and *toxR*^{C293SW76R} replicates that were subjected to immunoblotting in Fig. S5 A. The protein amount was analyzed by SDS-PAGE under standard non-reducing Laemmli buffer conditions and Kang staining (Kang *et al.*, 2002).

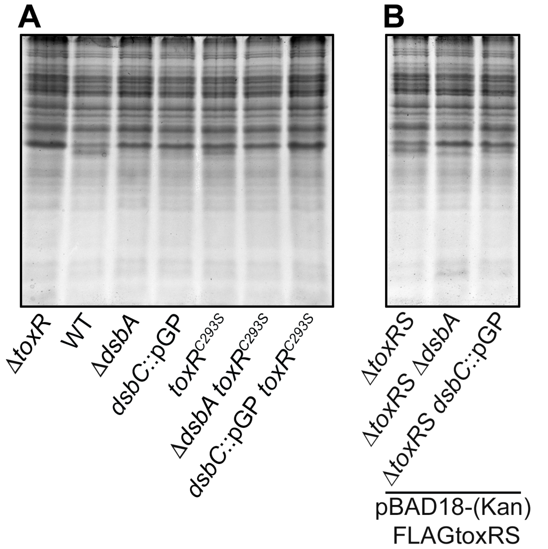

Figure S6 Protein amounts of strains used for the characterization of ToxR, OmpU and OmpT expression profiles. *V. cholerae* strains harboring *dsbA*, *dsbC* and/or *toxR* mutations were grown in M9 maltose minimal medium until the mid-log phase was reached. For Δ*toxRS* strains carrying FLAG*toxRS* on pBAD18-Kan or pBAD18 samples were taken after 2 h induction with 0.1% arabinose in the mid-log phase. **A** WCL of *V. cholerae* strains encoding for chromosomal mutants as indicated. **B** WCL of *V. cholerae* Δ*toxRS, dsbA*, and *dsbC* mutant strains carrying FLAG*toxRS* on pBAD18-(Kan). WCL were analyzed by SDS-PAGE under standard non-reducing Laemmli buffer conditions and Kang staining (Kang *et al.*, 2002) as loading controls for the immunoblot displayed in Fig. 6A-D.

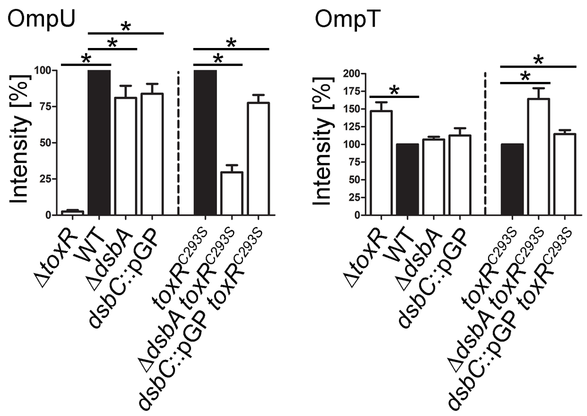

Figure S7 Dsb proteins influence ToxR inter- and intramolecular disulfide bond formation and its activity. The column bar graph displays the protein band intensities of OmpU and OmpT as a result of densitometric analysis of *V. cholerae* strains harboring *dsbA*, *dsbC* and/or *toxR* mutations which were grown in M9 maltose minimal medium until the mid-log phase was reached. WCLs of four biological replicates each with a technical replicate were loaded on SDS gels. Immunoblotting was performed under standard reducing Laemmli buffer conditions using anti-OmpU and anti-OmpT antibodies. OmpU/T band intensities (%) were readout using Image lab software (BIO-RAD) (see representative immunoblot Figure 6 B, C). The mean values with standard deviation are shown (n=6). The asterisks indicate significantly different means with P < 0.05 for the respective columns (white bars) each tested against the WT or the *toxR*^{C293S} mutant strain (black bars, intensities were defined as 100%) respectively using one-way ANOVA test, followed by Dunnett's post hoc test for multiple comparisons.

Kang, D., Gho, Y.S., Suh, M., and Kang, C. (2002) Highly sensitive and fast protein detection with coomassie brilliant blue in sodium dodecyl sulfate-polyacrylamide gel electrophoresis. *Bull. Kor. Chem. Soc.* **23**: 1511-1512.
